# Social representations of radical prostatectomy from the perspective of men undergoing surgery^*^

**DOI:** 10.1590/1980-220X-REEUSP-2025-0127en

**Published:** 2025-11-17

**Authors:** Bianca de Moura Peloso-Carvalho, Rogério Silva Lima, José Vitor da Silva, Eliza Maria Rezende Dázio, Murilo César do Nascimento, Silvana Maria Coelho Leite Fava

**Affiliations:** 1 Universidade Federal de Alfenas, Programa de Pós-graduação em Enfermagem, Alfenas, MG, Brazil.; 2 Universidade Federal de São Carlos, Departamento de Gerontologia, São Carlos, SP, Brazil.

**Keywords:** Prostatectomy, Prostatic Neoplasms, Qualitative Research, Social Representation, Oncology Nursing

## Abstract

**Objective:**

To understand the social representations of radical prostatectomy from the perspective of men undergoing surgery.

**Method:**

Qualitative, descriptive research, based on the theory of Social Representations. Sixty men diagnosed with prostate cancer and undergoing radical prostatectomy, who were assisted in a municipality in southern Minas Gerais, participated. Data collection took place between February and September 2022, through interviews and access to medical records. The characterization data were tabulated and presented in absolute and relative frequency and the qualitative data were transcribed and analyzed using the Collective Subject Discourse method.

**Results:**

Eleven Central Ideas were identified, associated with negative impacts on sexual and urinary functions and male identity; with neutrality, through satisfactory adaptation and positive perceptions: healing, relief and satisfaction; and with decision and confidence in the surgical procedure.

**Conclusion:**

Social representations revealed a complexity of experiences related to satisfaction, adaptation and dissatisfaction with post-surgical results.

## INTRODUCTION

Prostate cancer is evidenced as the most common neoplasm in men in 112 countries, which represents 15% of all cancer types. Global demographic changes and life expectancy rise suggest the number of new cases per year will increase from 1.4 million in 2020 to 2.9 million by 2040^(1)^. Nationally, it is the most common type, without considering non-melanoma skin tumors^(2)^.

Regarding therapy, radical prostatectomy is the most appropriate procedure for the treatment of clinically localized prostate cancer, considering disease control and cancer mortality. This procedure consists of the total resection of the prostate, seminal vesicles, lymph nodes and other affected pelvic structures, and can be performed openly, laparoscopically or robotically^(3)^. Regarding the number of surgeries performed per year, the Brazilian Society of Urology projected that 21,219 surgical procedures will be performed for the treatment of prostate cancer by 2025^(4)^.

This surgical procedure may present, as undesirable consequences, urinary incontinence and erectile dysfunction, which are adverse effects that contribute to the impact of surgery on quality of life^(5)^, so that the representations attributed to health, masculinity, functional losses and self-perception influence the way men understand and experience these impacts, culminating in a better or worse coping with illness, which demands understanding by health professionals^(6,7)^.

The framework of the Theory of Social Representations offers a theoretical framework that allows for the capture of symbolic and collectively constructed aspects, through an approach to the consensual and reified universe. In this regard, this framework prompts the apprehension of how men understand and deal with their illness, considering that the phenomena are processed as singular and subjective events, at the same time that they are socially elaborated and shared^(7,8)^.

Qualitative studies that seek to explore the male experience in relation to prostatectomy in light of the theory of Social Representations are still incipient at the national and international level, given the epidemiological context of prostate cancer and the number of surgical procedures performed. Given the above, this study aims to understand the social representations of radical prostatectomy from the perspective of men undergoing surgery.

Nursing, as a central area in the care of men who undergo prostatectomy, can directly benefit from the findings of this research, as the subjective repercussions of the surgery, in addition to the clinical aspects, can support the implementation of care and educational interventions that consider emotional, relational and identity aspects of the postoperative period, with a view to contributing to the advancement of nursing science in this care and research scenario.

## METHOD

### Design of Study

This is descriptive research, with a qualitative approach, based on the theoretical framework of the Theory of Social Representations and the methodological framework of the Collective Subject Discourse (CSD)^(7,8)^.

Social Representations, as common sense, are present in opinions, speeches, positions, messages and images in the media, being considered ways of understanding and communication, situated between the perceptions learned in everyday life and the meanings attributed by people^(8)^.

The CSD technique allows the access to Social Representations, in which individual expressions are grouped into categories, containing individual and collective opinions, experiences and life stories. To construct the CSDs, the researcher must work with the following methodological figures: Key Expressions, which are continuous or discontinuous excerpts of the discourse, which correspond to the answers regarding the guiding question; Central Ideas, which are the expressions that describe in a more synthetic way the meaning or meanings of the Key Expressions; and the CSD, which is the union of the Key Expressions related to the Central Ideas that have the same meaning. These expressions must be gathered, edited and written in the first person singular to configure the CSD^(9)^.

The conduction of this study and the construction of its report complied with the principles of *Consolidated Criteria for Reporting Qualitative Research-* COREQ^(10)^.

### Study Local

The research was conducted at a highly complex oncological center, which serves 26 municipalities in the south of Minas Gerais. The institution has six floors, the first being dedicated to multidisciplinary offices and radiotherapy, the second to chemotherapy and hormone therapy, and the remaining for hospitalization, with 84 equipped beds. It has a team of doctors, nurses, pharmacists, psychologists, social workers, and nutritionists who work in an integrated manner.

### Population and Selection Criteria

The inclusion criteria adopted were men diagnosed with prostate cancer (ICD C61), over 18 years of age, who were undergoing oncological treatment or follow-up, who underwent radical prostatectomy surgery, regardless of the technique used in the procedure and the time of its performance. Exclusion criteria were men who had some difficulty in understanding the research and/or participating in the study and that, through the application of the Mental Assessment Questionnaire^(9)^, got fewer than seven of the ten questions on the instrument right.

Seventy-one men were contacted. Of these, seven refused to participate in the study, one was excluded due to difficulties in understanding the research and three because, even though their medical records stated that they had undergone prostatectomy, they denied having the procedure performed. Thus, 60 men composed this study, constituting a convenience sample.

### Data Collection

The period allocated for data collection comprised the months of February to September 2022, in which, under the supervision of the outpatient clinic secretaries, the first author of the work, who has a master’s degree in nursing, with experience in qualitative data collection, accessed the medical records of potential participants, who were in the waiting room of the oncology outpatient clinic, for the survey of men undergoing prostatectomy surgery.

With this data in hand, the researcher cordially approached the men in the waiting room and invited them to join her in a private room near the offices, to ensure a safe environment and prevent interruptions and noise. If the men were accompanied, the companions were invited to participate only in this initial moment, that is, the invitation to participate in the study and clarifications about the research objectives and procedures. After the participant’s consent, companions were invited to wait in the waiting room until the end of data collection. In the private room, only the researcher and the participant remained, this being the first contact. The Mental Assessment Questionnaire was applied^(11)^ and depending on the result, the collection continued with the interview.

The interview form consisted of questions regarding the sociodemographic and clinical characteristics of the participants and guiding questions. Regarding characteristics, the questions referred to age, marital status, who the person lives with, education level, approximate monthly income, occupation, treatment performed, year of prostatectomy surgery, and the guiding question: Please tell me, what does prostate removal surgery mean to you?

It should be noted that the interviews were conducted solely based on the guiding question, according to the CSD method^(9)^.

An interview was conducted with each participant, but the transcription of the data was not returned to them. The logistics and dynamics of the service do not allow compliance with this item provided by COREQ, since each participant has a treatment dynamic and subsequent return to the outpatient clinic.

The statements were audiorecorded on two cell phones, a Xiaomi^®^ and a Samsung^®^, with a recorder application, simultaneously. The interviews lasted an average of approximately 30 minutes. Data collection from medical records was conducted with the assistance of the archives department team who organized the participants’ records. The procedure was supervised by professionals in the sector and was intended to obtain data regarding the date of the surgery and the treatments performed.

Data collection took place during the COVID-19 pandemic; therefore, the following biosafety measures were adopted: the interview locations were ventilated, so both participants and the researcher wore masks and maintained a physical distance of 1.5 m; in addition, both participants’ hands were disinfected with hand sanitizer at the beginning and end of each interview.

### Data Analysis and Treatment

The data corresponding to the sociodemographic and clinical characterization were tabulated in the Microsoft Excel^®^ software 2010 and presented in text format, using absolute and relative distributions. Qualitative data were transcribed in full using the Microsoft Word 2010 text editor, respecting orality.

Qualitative data were explored through extensive and rigorous vertical and horizontal reading of the individual speeches, with the use of the Discourse Analysis Instrument 1 – DAI1 and the Discourse Analysis Instrument 2 – DAI2 being chosen, which enable a systematic organization of the Key Expressions and Central Ideas, to allow the interpretative fidelity of the speeches, increasing transparency in the analytical process^(12)^.

After analyzing the textual data and Key Expressions, the equal, similar and complementary Central Ideas were grouped. Next, the emerging meanings and the participants who contributed to each representation were identified; and, finally, the Collective Subject Discourses were developed, in which three researchers with mastery of the method participated in this stage of analysis, ensuring analytical rigor and fidelity to the narratives.

### Ethical Aspects

The research began with approval of the project by the Research Ethics Committee of the Universidade Federal de Alfenas, with the Opinion and CAAE number: 5.131.466.

After understanding the objective of the study and accepting it, participants received the Informed Consent Form, containing clear and concise information about the research, objectives, procedures, possible benefits and risks, in addition to the ethical aspects applied to research with human beings, in accordance with the National Health Council (CNS) Resolution 466/12.

After signing the agreement to participate, one copy of the term was given to the participant and the other remained with the researcher. The participants’ identity was preserved and their personal names were replaced by codes with the initial E for interviewee in Portuguese (*entrevistado*), followed by the number in the sequence in which they were approached by the researcher, such as: E1, E2, E3, successively.

## RESULTS

Of the 60 men who made up the study population, in terms of age, the range was 51 to 87 years, the average was 68.7 years, with 41.67% (n = 25) aged between 60 and 69 years and 40% (n = 24) between 70 and 79 years. There was a predominance among those who declared living only with their wife, 51.67% (n = 31), and having completed elementary school, 36.67% (n = 22).

Regarding marital status, income, and occupation, these characteristics were analyzed before and after prostatectomy surgery. Regarding marital status before surgery, the majority (90%) of men were married or in a stable relationship (n = 54). After the surgery, only two married men became widowers, while the others remained in the same condition.

Regarding approximate monthly income, 36.67% (n = 22) declared receiving a minimum wage per month. After prostatectomy, 80% (n = 48) observed no change in their income, while 13.33% (n = 8) reported worsening and 6.67% (n = 4) indicated improvement.

Regarding occupation, 61.67% (n = 37) were retired before surgery and remained in that condition. Among those who worked, 38.33% (n = 23), 5% (n = 3) retired after surgery, 1.67% (n = 1) became unemployed, and 1.67% (n = 1) changed profession, going from bricklayer to farmer.

**Figure 1:**
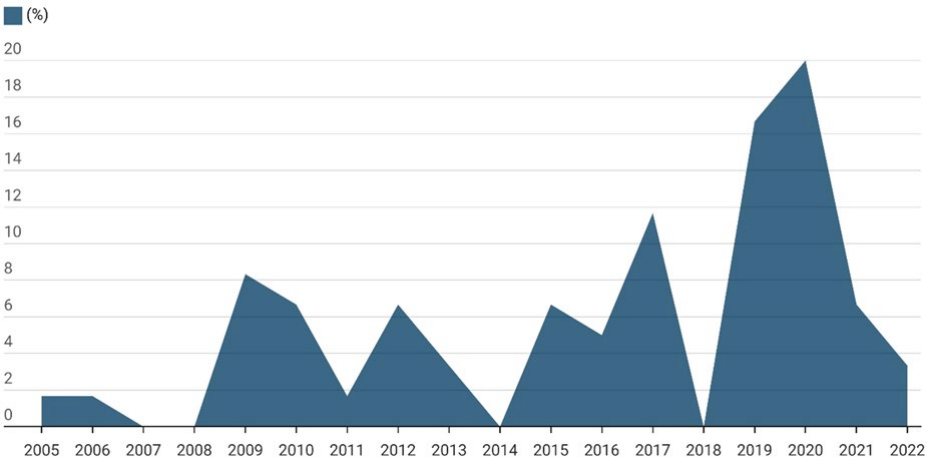
Temporal distribution of years when study participants underwent prostatectomy. Alfenas, MG, 2025 (n = 60).

Regarding clinical characterization, surgeries were performed between 2005 and 2022, with the most significant distribution, 46.67% (n = 28), in the last four years, as shown in Figure 1.

From the data analysis, 11 Collective Subject Discourses were identified, which were grouped into five discussion topics, according to similar meanings. Below, the fragments of the speeches are presented, indicating the frequency related to the number of participants who contributed to each speech, as well as the coded identification of these participants.

### 1) Positive perceptions: healing, relief and satisfaction with surgery (CSD A and CSD E)

*CSD A – It was good, great,* 36.67% (n = 22)

*Look, for me it was very good, really great, because I didn’t suffer anymore, it ended everything bad that I was feeling (…) I felt a lot of pain when urinating and I also felt tired for no reason, discouraged (…) Surgery takes that out of our heads, that problem that we have but can’t see (E01, E04, E06, E12, E15, E16, E17, E22, E23, E25, E29, E33, E35, E40, E47, E48, E50, E51, E53, E54, E56 and E60)*.

*CSD E – Remove the problem, don’t die, cure the cancer*, 38.33% (n = 23)

*It means that you removed the part that had the problem. If I hadn’t had my prostate removed, I would be underground today (…) I think it cured the cancer. I feel like I was born again (…) It is something that God gave us to be cured and not die before, it is a blessing. It was a relief for me, I started feeling much better (E04, E06, E10, E13, E14, E15, E17, E19, E21, E22, E26, E37, E38, E39, E45, E47, E48, E50, E52, E54, E56, E57 and E60).*

### 2) Decision and confidence: necessary surgical procedure (CSD F and CSD K)

*CSD F – Something that had to be done, otherwise the tumor would grow and get worse*, 41.67% (n = 25)

*Look, it was a necessary intervention due to a malignant tumor, cancer. I chose to have the surgery (…) because if I left it, it would get worse and then worse, little by little it takes over (…) it means taking care of your health (…) the doctor told me: “If you have surgery, it’s more secure” (E04, E06, E10, E13, E14, E15, E17, E19, E21, E22, E26, E27, E36, E37, E38, E39, E45, E47, E48, E50, E52, E54, E56, E57 and E60)*.

*CSD K – Something that could have already been done,* 3.33% (n = 2)

*I could have had the surgery a long time ago, but I thought it wasn’t that important, I didn’t know. I suffered for about five, six years taking medication, I could have felt better sooner, but it was still good (E22 and E56).*

### 3) Standardization: adaptation and conformity (SCD C and SCD I)

*SCD C – Nothing has changed, normal life*, 28.33% (n = 17)

*Regarding the surgery, it didn’t harm me at all, my life continues as normal (…) I didn’t feel any pain, I didn’t feel anything. (…) My sexual function continues to work (…), but I know that for many men this is not the case. Besides, I was lucky not to have had any problems with my urine (…) And there are people who need to do chemotherapy, radiotherapy, and I didn’t (…) (E01, E02, E04, E07, E10, E18, E20, E21, E31, E41, E43, E44, E45, E48, E50, E51 and E53).*

*SCD I – Something you get used to, there’s no point in regretting it*, 6.67% (n = 4)

*Look, it’s been two years since I had the surgery and today I’m a little more used to the situation (…) you need to take care of your prostate when you’re younger, because after things happen, there’s no point in complaining (…) I’m not shy or ashamed to talk about it with anyone. Many people may make fun of it, but I don’t care. What matters is my health (…) and I have my wife by my side (E12, E18, E30 and E52).*

### 4) Negative impacts: sexual function, urinary function and male identity (SCD B, SCD D and SCD H);

*SCD B – Impaired sexual function*, 40% (n = 24)

*If I had known I would end up like this, I wouldn’t have had the surgery (…) because surgery has something bad, regarding is sexual intercourse (…) I think doctors should explain the post-operative period to us (…) I feel like something is missing, it’s as if I were mutilated (…) surgery takes away pleasure and much of what a man exercises as a human being, as a male, his virility is compromised. I no longer have a relationship with my wife, I am worthless and this is unpleasant (E01, E02, E03, E04, E08, E09, E15, E16, E19, E23, E24, E26, E29, E30, E31, E32, E34, E36, E39, E51, E52, E58, E59 and E60).*

*SCD D – Urinary problems*, 10% (n = 6)

*(…) After the operation (…) my urine is loose, I use disposable diapers all the time. If I stay still, my urine is controlled, but if I start coughing or straining, the pee starts to escape little by little. This happens because they put in the probe, which stays in for 15 days and dilates. Today, my urine is like a shower, there is no toilet that can handle it, I even have to pee sitting down (…) there is no cure for loose urine (E02, E06, E15, E34, E38 and E42).*

*SCD H – Changes, various sequelae and negative consequences*, 23.33% (n = 14)

*Having my prostate removed for a man like me, who has always lived with it, is not because of chauvinism, but it is sorely missed. You feel like a despised man, because as a man you have no action (…) after I had the operation I am nobody anymore, the fact of wearing diapers finished me off. If we could do a prostate implant it would be very good for men, because when you remove the prostate it ruins the man (…) The surgery saved my life, but I am not living (E03, E11, E12, E14, E17, E30, E38, E39, E41, E46, E49, E55, E58 and E60).*

### 5) Biopsychosocial challenges: suffering, worry and shock (SCD G and SCD J)

*SCD G – Suffering, very bad, difficult, unpleasant and sad experience*, 15% (n = 9)

*The surgery itself is unpleasant, it is frustrating. We go through difficult times. We feel bad because it cuts us and we feel a lot of pain for up to five days. For me it was just suffering, I have nothing good to say (…) it’s sad, I had to see a psychologist, because the name cancer is already scary. I think there should be greater psychological preparation (E12, E19, E20, E30, E34, E39, E45, E51 and E55).*

*SCD J – Fright, worry, shock*, 6.67% (n = 4)

*I was very worried, because everyone says, all men say, that prostate removal ends your sex life! It was a huge shock, at first it was a fright (…) I had a hard time getting back to my place, I was a bit lost (E19, E29, E30 and E39).*

**Figure 2:**
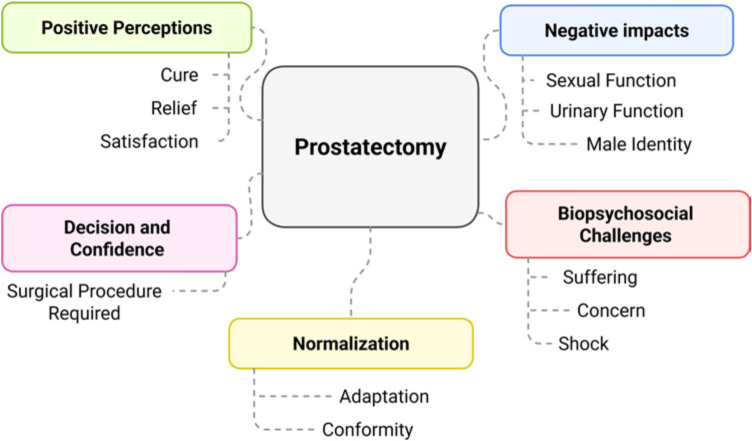
Summary illustration of social representations about prostate removal surgery based on men’s meanings through the collective subject discourse, Alfenas, MG, Brazil, 2025.

Figure 2 represents the panel of speeches, according to the groupings.

## DISCUSSION

Men perceived that surgery provided an end to suffering and symptoms that they classified as bad, according to SCD A. The perception of improvement in urinary symptoms and also in bone pain was evidenced in a study carried out with participants in an advanced stage of prostate cancer after orchiectomy surgery, demonstrating that surgical procedures are recognized as providing well-being^(13)^. However, improvement in lower urinary tract symptoms after prostatectomy does not occur immediately, as it can take six to 12 months, which highlights the importance of professional support for self-management of these symptoms until they stabilize^(14)^.

The positive perception of surgery was also attributed to the fact that surgery removes a problem that men thought they had but could not see, that is, by removing the cancer, the worries are also removed. The literature indicates that men on active surveillance expressed anxiety regarding cancer progression, as this therapeutic modality is not definitive^(15)^. Thus, it is understood that definitive treatment, such as surgical removal, may symbolize more adequate cancer control for some men.

In SCD E, surgery was seen as a procedure that removed a part of the body that was malfunctioning, that was not functioning well, and that could cause death, demonstrating a familiarity with cancer and its consequences through anchoring. The results of a study corroborate this discourse, since men, fearing the progression of the disease, saw the surgical option as the only way to eliminate this fear, cure cancer, and prolong life^(16)^.

Furthermore, surgery was seen as a divine blessing, as it ended suffering, brought healing, and prevented death. In line with these results, those who opted for surgical prostate removal reported relief at ridding their bodies of cancer and that God had blessed them^(15)^.

In SCD F, prostatectomy was seen as a necessary intervention for a malignant tumor, which could slowly worsen and spread. To represent the decision to undergo prostatectomy, they used technical terms, such as “malignant tumor,” while also anchoring meanings when describing the consequences of cancer. This result is consistent with the findings of a study in which participants recognized the need for the surgical procedure, even if it meant losses related to sexual and urinary functions, and that faced with this dilemma, the option for life was greater than the negative consequences arising from the surgery^(16)^.

In SCD K, participants claimed to be unaware of the importance of prostatectomy and regretted not having undergone it sooner. A study that evaluated men’s experiences and the impact on their well-being showed that those who underwent radiotherapy and had a recurrence of prostate cancer believed that total prostate removal would have prevented this problem^(17)^.

This SCD confirms the need for healthcare professionals to provide personalized information on therapeutic options, their risks and benefits^(18)^, respecting the principles of health literacy, with language that facilitates understanding, promoting autonomy in the decision-making process regarding therapy, as the occurrence of side effects and recurrence of cancer can lead to doubts about the effectiveness of the chosen option and trust in health professionals^(18,19)^.

In SCD C, men reported that prostatectomy did not cause any sequelae or changes in their lives. The effectiveness of treatment, the absence of pain, and the preservation of sexual and urinary function may be related to these representations, since, as they say, these are consequences that may affect others in their environment.

Similar perceptions were found in a study in which the participant reported that he felt very lucky, because even though he had erectile dysfunction, he was able to maintain sexual relations through medication, unlike other men he knew^(15)^. In another study, the self-perception of “someone who had cancer,” associated with little impairment in general well-being, was assumed by younger and more sexually active men^(5)^. Thus, it is understood that maintaining sexual function, even with the help of therapy, makes men represent surgery as something that had little or no impact on their lives.

In SCD I, for participants, there is no point in regretting having a prostatectomy, as it is necessary to take care of the prostate when one is younger, thus avoiding cancer as a consequence. Research results found that participants reported that harmful habits, such as alcohol and tobacco consumption, caused cancer, and that in youth, people do not think about the consequences of these habits^(20)^.

In another study, men 10 years after prostatectomy reported that their best advice to other men was about the importance of health care. This experience, according to the authors, was a way for men to connect with collective masculinity^(15,21)^. Therefore, experiences with prostate cancer and surgery have led men to value care related to measures to prevent the disease.

For the participants in this study, undergoing a prostatectomy may be a reason for people to “mock” due to the after-effects of the surgery; however, they did not feel embarrassed by this reality. The stigma of prostate cancer that permeates the symbolic universe of some men can lead to vulnerability, causing men who have undergone prostatectomy to hide their struggles and keep the adverse effects of the surgery secret, culminating in social isolation^(22)^.

That said, it is understood that men need to overcome the embarrassment that permeates beliefs about masculinity, so that they can obtain the support they need to face cancer and the consequences arising from therapeutic procedures.

The weight of the representation of the loss of sexual function, evidenced in SCD B, led men to question the relevance of the clarifications provided by health professionals, which could guide them to not undergo surgery. Similar results were found in an article in which men regretted having a prostatectomy after gaining knowledge from reading scientific texts, as they understood that by postponing the procedure they could have a longer sexual life^(15)^. This discourse recognizes the burden that the loss of sexual function represents, since knowledge of this problem could guide them to not undergo surgery.

For the participants, prostatectomy was perceived as a “mutilation,” a representation that anchors the removal of a body part and its function. Research results showed that changes in erectile function made men feel unable to fulfill their duty as husbands in the couple’s sexual relationship^(16)^. That said, wives should be included in the care process so that they can discuss, together with their spouse and health professionals, the adverse effects of prostatectomy^(23)^.

Given this reality, the importance of pre-rehabilitation has been advocated, which consists of a multidisciplinary approach before the surgical procedure, which in prostatectomy consists of addressing potential side effects before they manifest after surgery. This approach aims to improve a person’s functional capacity, psychological resilience and overall health, alleviating the negative impact of surgery on quality of life. Interventions used in this phase include: physical activity, peer support (e.g., a group of men with cancer), pelvic floor muscle training, nutritional guidance, and administration of phosphodiesterase-5 inhibitors^(24)^. This approach is essential for men to better re-evaluate the adverse effects of prostatectomy, motivating them to seek resources to mitigate these effects.

Regarding SCD D, participants said that after surgery they noticed a loss of urinary control, requiring changes in habits, such as toilet positioning and diaper use. In this discourse, participants used the image of a shower to anchor representations regarding the way they understand the current urinary pattern.

Similar results were found in studies in which men reported that incontinence affects their sleep, as diapers and pads are not fully efficient in controlling urinary volume during the night, which makes it difficult to sleep^(15)^, and causes men to avoid social occasions^(25)^, feel embarrassed when using public restrooms^(26)^, have trouble traveling, worry about having bad odor, and restrict their fluid intake^(25,27)^. Furthermore, the use of diapers was pointed out as something that depersonalized them, making men feel like children^(14)^.

In view of this context, we understand the impacts that the loss of sphincter control can represent, altering the way men live, behave, and establish their social relationships.

In this context, as a strategy for monitoring men with incontinence, Brazilian researchers developed the IUProst® application, which is considered a technology that can favor the care provided by nurses, since this application offers information on changes in lifestyle habits and the performance of exercises to strengthen the pelvic muscles, allowing the exercises to be performed remotely, through a smartphone^(28)^.

In SCD H, the prostate implant was represented as an alternative to replacing the removed prostate. It is inferred that the prostate is represented as an organ associated with erectile capacity, so that an implant would allow them to resume this function.

For the collective subject, the surgical procedure was represented as something to save life, however, the negative impact of the consequences changed the perception, demonstrating that the meaning of living is linked not only to not dying, but to how one lives.

In this context, nursing must seek innovative care approaches that contribute to improving quality of life. Evidence of collaborative care demonstrates that professional nursing skills, combined with scientific research, focusing on individual needs, were effective in developing personalized care plans for men after prostatectomy, since emotional well-being, better self-care capacity and quality of life and reduction of postoperative complications were evidenced^(29)^.

In SCD G, the surgery was perceived as something unpleasant and frustrating, which made him go through difficult times, causing physical and psychological discomfort, resulting from the procedure and also from the word cancer. A study shows that the social representation of cancer for adults undergoing cancer treatment initially permeates meanings related to illness, sadness, and death, with sadness being presented as a negative feeling when faced with the possibility of becoming ill and death as a direct relationship linked to the cancer diagnosis^(7)^. In these circumstances, there is a need for dialogue and psychological support, so that men can face treatment with more peace of mind.

Study results corroborate these findings, considering that men undergoing treatment for prostate cancer deal with ongoing psychological problems related to cancer and its treatment, which has impacted their self-esteem and relationships. They also pointed out that seeking support for their psychosocial problems is challenging, as discussing these issues is embarrassing for them^(30)^.

In SCD J, the representations that permeate the male consensual universe referred to concern about the loss of sexual function, as observed in the literature, in which concerns about possible unwanted results caused men to be uncertain about which treatment they should choose, since, at the same time as they perceived better results through surgery, they were uncertain about the risks of urinary and sexual dysfunction, this being the central factor in the decision-making process^(17)^.

Thus, it is understood that representations about the undesirable effects of prostatectomy permeate the male consensual universe, causing concerns, which can guide the therapeutic decision-making process.

Moreover, they reported that it was a shock and a scare. It infers that men represented surgery as something that shakes them, that takes them out of their place, and hinders their return to “normal”. This place can symbolize their identity as healthy men, in control of their bodies and functions. The importance of men being informed about the surgical procedure is reiterated, given that most participants felt they did not have the necessary information to manage long-term side effects, and that they did not know how they would return to “normal,” so they were unaware of the resources available to manage their ongoing problems^(30)^.

As limitations of this study, it is considered that it was not possible to identify situations of disease remission, the type of laparoscopic or open surgical approach and preservation of neurovascular bundles, as well as data related to urinary incontinence and sexual dysfunction, which could contribute to a better characterization of the participants. Furthermore, the interviews were not returned to the participants due to the flow of care at the cancer center.

As contributions to practice, the results revealed the importance of health literacy for understanding the guidelines, which contributes to men’s co-participation in therapeutic decisions, and also the participation of partners/wives in guidance on prostatectomy and its adverse effects. For these guidelines to be effective, spaces for dialogue must be created that allow men to overcome the barriers of embarrassment and obtain the support they need.

Personalized, innovative strategies, supported by self-care and current scientific evidence, can help alleviate suffering, improve physical functions related to sexuality and urinary function, and improve understanding of the disease and treatments.

## CONCLUSION

The analysis of social representations revealed that, for some men, radical prostatectomy was seen as a beneficial and curative procedure, a necessary health care, demonstrating the benefits and satisfaction with the treatment. In contrast, other discourses expressed prostatectomy as an experience related to suffering, impairments in sexual function, virility, urinary control, and marital relationships. The absence of the prostate was lamented, and they feel that surgery saved their lives, but that they are not living.

It is suggested that studies be carried out in other sociocultural contexts and that the postoperative period, as well as the treatment and follow-up phases, be demarcated.

## Data Availability

The entire dataset that supports the findings of this study has been made available in SciELO Data and can be accessed at https://doi.org/10.48331/SCIELODATA.AJLTDE.
