# Supplementary material for: Social representations of radical prostatectomy from the perspective of men undergoing surgery
Source: Rev Esc Enferm USP. 2025 Nov 17;59:e20250127. doi: 10.1590/1980-220X-REEUSP-2025-0127en (PMC12622884; doi:10.1590/1980-220X-REEUSP-2025-0127en)
Supplement: Translation

# Translation: Representações sociais da prostatectomia radical na perspectiva de homens submetidos à cirurgia^*^

**DOI:** 10.1590/1980-220X-REEUSP-2025-0127pt

**Authors:** Bianca de Moura Peloso-Carvalho, Rogério Silva Lima, José Vitor da Silva, Eliza Maria Rezende Dázio, Murilo César do Nascimento, Silvana Maria Coelho Leite Fava

**Affiliations:** 1 Universidade Federal de Alfenas, Programa de Pós-graduação em Enfermagem, Alfenas, MG, Brasil.; 2 Universidade Federal de São Carlos, Departamento de Gerontologia, São Carlos, SP, Brasil.

**Keywords:** Prostatectomia, Neoplasias de la Próstata, Pesquisa Qualitativa, Representação Social, Enfermagem Oncológica

## Abstract

**Objetivo:**

Compreender as representações sociais da prostatectomia radical na perspectiva de homens submetidos à cirurgia.

**Método:**

Pesquisa qualitativa, descritiva, fundamentada na teoria das Representações Sociais. Participaram 60 homens, diagnosticados com câncer de próstata, submetidos à prostatectomia radical, atendidos em um município do Sul de Minas Gerais. Coleta de dados entre fevereiro e setembro de 2022, por meio de entrevistas e do acesso aos prontuários. Os dados de caracterização foram tabulados e apresentados em frequência absoluta e relativa e os qualitativos, transcritos e analisados pelo método do Discurso do Sujeito Coletivo.

**Resultados:**

Identificaram-se 11 Ideias Centrais, associadas aos impactos negativos nas funções sexuais, urinárias e na identidade masculina, à neutralidade, mediante a adaptação satisfatória e às percepções positivas: cura, alívio e satisfação, além da decisão e confiança no procedimento cirúrgico.

**Considerações Finais:**

As representações sociais revelaram uma complexidade de experiências, relacionadas à satisfação, adaptação e à insatisfação com os resultados pós-cirúrgicos.

## INTRODUÇÃO

O câncer de próstata é evidenciado como a neoplasia mais comum em homens em 112 países, o que representa 15% entre os tipos de câncer. Mudanças demográficas mundiais e aumento da expectativa de vida sugerem que o número de casos novos por ano aumentará de 1,4 milhão em 2020 para 2,9 milhões até 2040^(1)^. No âmbito nacional, configura-se como o mais incidente, sem considerar os tumores de pele não melanoma^(2)^.

Referente à terapêutica, a prostatectomia radical é o procedimento mais adequado para o tratamento do câncer de próstata clinicamente localizado, considerando-se o controle da doença e a mortalidade pelo câncer. Esse procedimento consiste na ressecção total da próstata, vesículas seminais, linfonodos e outras estruturas pélvicas acometidas, podendo ser realizado por via aberta, laparoscópica e robótica^(3)^. Em relação ao número de cirurgias realizadas por ano, a Sociedade Brasileira de Urologia projetou para 2025 a realização de 21.219 procedimentos cirúrgicos para o tratamento do câncer de próstata^(4)^.

Este procedimento cirúrgico pode apresentar, como consequências indesejadas, a incontinência urinária e a disfunção erétil, que são efeitos adversos que contribuem para o impacto da cirurgia na qualidade de vida^(5)^, de modo que as representações atribuídas à saúde, à masculinidade, às perdas funcionais e à autopercepção influenciam o modo como os homens entendem e vivenciam esses impactos, culminando em um melhor ou pior enfrentamento do adoecimento, o que demanda a compreensão pelos profissionais de saúde^(6,7)^.

O referencial da Teoria das Representações Sociais oferece um arcabouço teórico, que permite captar aspectos simbólicos e coletivamente construídos, por meio da aproximação com o universo consensual e reificado. Neste entendimento, esse referencial propicia compreender como os homens entendem e lidam com o seu adoecimento, considerando-se que os fenômenos se processam como eventos singulares e subjetivos, ao mesmo tempo que são socialmente elaborados e compartilhados^(7,8)^.

Estudos qualitativos que buscam explorar a experiência masculina em relação à prostatectomia à luz da teoria das Representações Sociais ainda são incipientes no âmbito nacional e internacional, em face do contexto epidemiológico do câncer de próstata e do número de procedimentos cirúrgicos realizados. Diante do exposto, esse estudo objetiva compreender as representações sociais da prostatectomia radical na perspectiva de homens submetidos à cirurgia.

A Enfermagem, como área central no cuidado ao homem que se submete à prostatectomia, pode se beneficiar diretamente dos achados desta pesquisa, na medida em que as repercussões subjetivas da cirurgia, para além dos aspectos clínicos, podem subsidiar a implementação de intervenções assistenciais e educativas que considerem aspectos emocionais, relacionais e identitários do pós-operatório, com vistas a contribuir para o avanço da ciência da Enfermagem neste cenário de cuidados e de pesquisa.

## MÉTODO

### Tipo de Estudo

Trata-se de pesquisa descritiva, de abordagem qualitativa, fundamentada no referencial teórico da Teoria das Representações Sociais e metodológico do Discurso do Sujeito Coletivo (DSC)^(7,8)^.

As Representações Sociais, enquanto senso comum, estão presentes nas opiniões, nos discursos, nos posicionamentos, nas mensagens e nas imagens na mídia, sendo consideradas maneiras de compreensão e de comunicação, situando-se entre as percepções apreendidas pelo cotidiano e os significados atribuídos pelas pessoas^(8)^.

A técnica do DSC possibilita o acesso às Representações Sociais, em que expressões individuais são agrupadas em categorias, contendo opiniões, experiências e vivências individuais e coletivas. Para que se construa os DSC, o pesquisador deve trabalhar com as seguintes figuras metodológicas: as Expressões Chave, que são trechos contínuos ou descontínuos do discurso, que correspondem às respostas referentes à pergunta norteadora; as Ideias-Centrais, que são as expressões que descrevem de maneira mais sintética o sentido ou os sentidos das Expressões Chave; e o DSC, que é a união das Expressões Chave relacionadas às Ideias Centrais que possuem o mesmo sentido. Essas expressões devem ser reunidas, editadas e redigidas na primeira pessoa do singular para que se configure o DSC^(9)^.

A condução deste estudo e a construção do seu relatório cumpriram os princípios do *Consolidated Criteria for Reporting Qualitative Research-* COREQ^(10)^.

### Local do Estudo

A pesquisa foi conduzida em um centro de alta complexidade oncológica, que atende a 26 municípios do Sul de Minas Gerais. A instituição é composta por seis andares, sendo o primeiro destinado aos consultórios multiprofissionais e à radioterapia, o segundo à quimioterapia e hormonioterapia e os demais para internação, com 84 leitos equipados. Dispõe de equipe composta por médicos, enfermeiros, farmacêuticos, psicólogos, assistentes sociais e nutricionistas que atuam de forma integrada.

### População do Estudo e Critérios de Seleção

Adotaram-se como critérios de inclusão homens diagnosticados com câncer de próstata (CID C61), maiores de 18 anos, que estavam em tratamento ou acompanhamento oncológico, que se submeteram à cirurgia de prostatectomia radical, independente da técnica utilizada no procedimento e do momento de sua realização. Os critérios de exclusão foram homens que apresentassem alguma dificuldade na compreensão da pesquisa e/ou na participação no estudo e que, mediante a aplicação do Questionário de Avaliação Mental^(9)^, acertassem menos que sete, das dez perguntas do instrumento.

Foram contactados 71 homens. Destes, sete recusaram-se a participar do estudo, um foi excluído devido a dificuldades em compreender a pesquisa e três porque, mesmo constando em seus prontuários que se submeteram à prostatectomia, negaram a realização do procedimento. Dessa maneira, 60 homens compuseram este estudo, constituindo-se uma amostra por conveniência.

### Coleta de Dados

O período destinado à coleta de dados compreendeu os meses de fevereiro a setembro de 2022, em que, sob supervisão das secretárias do ambulatório, a primeira autora do trabalho, mestra em enfermagem, com experiência em coleta de dados qualitativos, acessou os prontuários dos possíveis participantes, que aguardavam na sala de espera do ambulatório de oncologia, para realizar o levantamento dos homens submetidos a cirurgia de prostatectomia.

De posse desses dados, a pesquisadora abordava cordialmente os homens na sala de espera e os convidava para acompanhá-la em uma sala privativa próxima aos consultórios, de modo a garantir um ambiente seguro e prevenir interrupções e ruídos. Caso os homens estivessem com acompanhantes, estes eram convidados para participar apenas desse momento inicial, ou seja, o convite para participar do estudo e os esclarecimentos sobre os objetivos e os procedimentos da pesquisa. Após a anuência do participante, os acompanhantes eram convidados a aguardar na sala de espera o término da coleta de dados. Na sala privativa, permaneceram apenas a pesquisadora e o participante, sendo este o primeiro contato. Foi aplicado o Questionário de Avaliação Mental^(11)^ e a depender do resultado, dava-se sequência à coleta com a realização da entrevista.

O formulário de entrevista era composto por questões referentes às características sociodemográficas e clínicas dos participantes e questões norteadoras. Quanto às características, as questões referiram-se à idade, ao estado conjugal, com quem mora, à escolaridade, à renda mensal aproximada, à ocupação, ao tratamento realizado, ao ano de realização da cirurgia de prostatectomia e à questão norteadora: Me conte, por favor, o que significa a cirurgia de retirada da próstata para o senhor?

Destaca-se que as entrevistas foram conduzidas apenas com base na questão norteadora, conforme o método do DSC^(9)^.

Foi realizada uma entrevista com cada participante, sendo que a transcrição dos dados não retornou aos mesmos. A logística e a dinâmica do serviço não permitem o cumprimento desse item previsto pelo COREQ, uma vez que cada participante apresenta uma dinâmica de tratamento e conseguinte retorno ao ambulatório.

Os depoimentos foram gravados em áudio e registrados em dois aparelhos de celulares, um Xiaomi^R^ e um Samsung^R^, com aplicativo de gravador, de maneira concomitante. As entrevistas tiveram duração média de aproximadamente 30 minutos. A coleta de dados nos prontuários foi conduzida com auxílio da equipe do setor de arquivos que organizou os registros dos participantes. O procedimento foi supervisionado pelos profissionais do setor e destinou-se à obtenção de dados referentes à data de realização da cirurgia e aos tratamentos realizados.

A coleta de dados ocorreu durante o período pandêmico pela COVID-19; assim, foram adotadas as seguintes medidas de biossegurança: os locais das entrevistas eram ventilados, de modo que, tanto os participantes quanto a pesquisadora usaram máscaras e mantiveram distanciamento físico de 1,5 m; além disso, houve desinfecção das mãos de ambos, com álcool gel, no início de no final de cada entrevista.

### Análise e Tratamento dos Dados

Os dados correspondentes à caracterização sociodemográfica e clínica foram tabulados no programa Microsoft Excel^R^ 2010 e apresentados em formato de texto, utilizando-se distribuições absolutas e relativas. Os dados qualitativos foram transcritos na íntegra com o auxílio do editor de texto Microsoft Word 2010, obedecendo a oralidade.

Os dados qualitativos foram explorados por meio de extensa e rigorosa leitura vertical e horizontal dos discursos individuais, optando-se por utilizar o Instrumento de Análise do Discurso 1-IAD1 e o Instrumento de Análise do Discurso 2-IAD2, que permitem uma organização sistemática das Expressões Chave e das Ideias Centrais, a fim de possibilitar a fidelidade interpretativa dos discursos, ampliando a transparência no processo analítico^(12)^.

Após análise dos dados textuais e das Expressões Chaves, foram agrupadas as Ideias Centrais iguais, semelhantes e complementares. Em seguida, identificaram-se os significados emersos e os participantes que contribuíram com cada representação; e, por fim, os Discursos do Sujeito Coletivo foram elaborados, em que três pesquisadores com domínio no método participaram dessa etapa de análise, garantindo rigor analítico e fidelidade às narrativas.

### Aspectos Éticos

A pesquisa teve início mediante aprovação do projeto pelo Comitê de Ética em Pesquisa da Universidade Federal de Alfenas, com o n° do Parecer e CAAE: 5.131.466.

Após a compreensão do objetivo do estudo e aceitação, os participantes receberam o Termo de Consentimento Livre e Esclarecido, contendo informações claras e concisas sobre a pesquisa, objetivos, procedimentos, possíveis benefícios e riscos, além dos aspectos éticos aplicados à pesquisa com seres humanos, de acordo com a Resolução do Conselho Nacional de Saúde (CNS) 466/12.

Após a assinatura concordando com a participação, foi entregue uma via do referido termo ao participante e outra ficou com a pesquisadora. A identidade dos participantes foi preservada e seus nomes pessoais foram substituídos por códigos com a inicial E de entrevistado, seguido do número na sequência em que foram abordados pela pesquisadora, como: E1, E2, E3, sucessivamente.

## RESULTADOS

Dos 60 homens que compuseram a população do estudo, no que se refere à idade, o intervalo foi de 51 a 87 anos, a média foi de 68,7 anos e 41,67% (n = 25) tinham idade entre 60 a 69 anos e 40% (n = 24) entre 70 a 79 anos. Observou-se predominância entre aqueles que declaram morar somente com esposa, 51,67% (n = 31), e ter cursado o ensino Fundamental I Completo, 36,67% (n = 22).

No que tange ao estado civil, à renda e à ocupação, estas características foram analisadas antes e depois da realização da cirurgia de prostatectomia. Em relação ao estado civil antes da realização da cirurgia, a maioria dos homens era casado ou em união estável 90% (n = 54). Após a cirurgia, apenas dois homens casados tornaram-se viúvos, enquanto os demais permaneceram na mesma condição.

Quanto à renda mensal aproximada, 36,67% (n = 22) declararam receber mensalmente um salário mínimo. Após a prostatectomia, 80% (n = 48) não observaram mudanças em sua renda, enquanto 13,33% (n = 8) relataram piora e 6,67% (n = 4) indicaram melhora.

Em relação à ocupação, 61,67% (n = 37) estavam aposentados antes da cirurgia e permaneceram nessa condição. Entre os que trabalhavam, 38,33% (n = 23), 5% (n = 3) aposentaram após a cirurgia, 1,67% (n = 1) ficou desempregado e 1,67% (n = 1) mudou de profissão, passando de pedreiro para lavrador.

No que diz respeito à caracterização clínica, a realização das cirurgias ocorreu entre 2005 e 2022, com a distribuição mais expressiva, 46,67% (n = 28), nos últimos quatro anos, conforme a Figura 1.

**Figura 1:** Distribuição temporal dos anos de realização da prostatectomia pelos participantes do estudo. Alfenas, MG, 2025 (n = 60).

Da análise dos dados, foram identificadas 11 Discursos do Sujeito Coletivo, que foram agrupados em cinco tópicos de discussão, conforme significados semelhantes. A seguir, apresentam-se os fragmentos dos discursos, indicando a frequência relativa ao número de participantes que contribuiu com cada discurso, bem como a identificação codificada desses participantes.

### 1) Percepções positivas: cura, alívio e satisfação com a cirurgia (DSC A e DSC E)

*DSC A – Foi bom, uma beleza,* 36,67% (n = 22)

*Olha, pra mim foi muito bom, ótimo mesmo, porque eu não sofri mais, acabou com tudo que eu estava sentindo de ruim (…) sentia muita dor pra urinar e também ficava cansado à toa, desanimado (…) A cirurgia tira aquilo da cabeça, aquele problema que a gente tem, mas não consegue ver (E01, E04, E06, E12, E15, E16, E17, E22, E23, E25, E29, E33, E35, E40, E47, E48, E50, E51, E53, E54, E56 e E60)*.

*DSC E – Retirar o problema, não morrer, curar o câncer*, 38,33% (n = 23)

*Significa que retirou a parte que estava com problema. Se não tivesse retirado a próstata, hoje estaria debaixo da terra (…) acho que curou o câncer. Sinto que eu nasci de novo (…) É algo que Deus nos deu para ter cura e não morrer antes, é uma bênção. Foi um alívio para mim, comecei a me sentir muito melhor (E04, E06, E10, E13, E14, E15, E17, E19, E21, E22, E26, E37, E38, E39, E45, E47, E48, E50, E52, E54, E56, E57 e E60)*.

### 2) Decisão e confiança: procedimento cirúrgico necessário (DSC F e DSC K)

*DSC F – Algo que era preciso ser feito, se não o tumor crescia e piorava*, 41,67% (n = 25)

*Olha, foi uma intervenção necessária devido a um tumor maligno, um câncer. Optei por fazer a cirurgia (…) porque se deixasse aumentava e depois ficava pior, devagarzinho vai tomando conta da gente (…) significa cuidar da saúde (…) o médico me disse: “Se você fizer a cirurgia, é mais garantido” (E04, E06, E10, E13, E14, E15, E17, E19, E21, E22, E26, E27, E36, E37, E38, E39, E45, E47, E48, E50, E52, E54, E56, E57 e E60)*.

*DSC K – Algo que podia já ter sido feito,* 3,33% (n = 2)

*Podia ter feito a cirurgia há muito tempo, mas eu achava que não era tão importante, não sabia. Fiquei sofrendo por uns cinco, seis anos tomando remédios, podia ter me aliviado antes, mas mesmo assim foi bom (E22 e E56)*.

### 3) Normalização: adaptação e conformidade (DSC C e DSC I)

*DSC C – Não mudou nada, vida normal*, 28,33% (n = 17)

*Sobre a cirurgia, ela não me prejudicou em nada, minha vida continua normal (…) não senti dor, não senti nada. (…) A minha função sexual continua funcionando (…), mas sei que para muitos homens não é assim. Além disso, tive sorte de não ter tido problemas com a urina (…) E tem gente que precisa fazer quimioterapia, radioterapia, e eu não (…) (E01, E02, E04, E07, E10, E18, E20, E21, E31, E41, E43, E44, E45, E48, E50, E51 e E53)*.

*DSC I – Algo com o que se acostuma, não adianta lamentar*, 6,67% (n = 4)

*Olha, já faz dois anos desde que fiz a cirurgia e hoje estou um pouco mais acostumado com a situação (…) é preciso cuidar da próstata quando se é mais novo, porque depois que as coisas acontecem, não adianta ficar lamentando (…) não tenho acanhamento ou vergonha em comentar sobre isso com ninguém. Muitas pessoas podem zombar, mas eu não esquento não. O que importa é minha saúde (…) e tenho minha esposa ao meu lado (E12, E18, E30 e E52)*.

### 4) Impactos negativos: função sexual, função urinária e identidade masculina (DSC B, DSC D e DSC H);

*DSC B – Comprometimento na função sexual*, 40% (n = 24)

*Se eu soubesse que ficaria assim, eu não tinha feito a cirurgia (…) porque a cirurgia tem uma coisa que prejudica, que é na relação sexual (…) eu acho que os médicos deveriam explicar pra gente o pós-operatório (…) sinto que falta algo, é como se fosse mutilado (…) a cirurgia tira o prazer e muito daquilo que o homem exerce como ser humano, como macho, sua virilidade é comprometida. Não tenho mais relação com minha esposa, não valho nada e isso é desagradável (E01, E02, E03, E04, E08, E09, E15, E16, E19, E23, E24, E26, E29, E30, E31, E32, E34, E36, E39, E51, E52, E58, E59 e E60)*.

*DSC D – Problemas urinários*, 10% (n = 6)

*(…) Depois que operei (…) minha urina fica solta, uso fralda descartável direto. Se eu fico parado, minha urina fica controlada, mas se começo a tossir ou fazer força, o xixi vai escapando aos pouquinhos. Isso acontece porque colocaram a sonda, que fica por 15 dias e dilata. Hoje, minha urina parece um chuveiro, não tem vaso sanitário que aguente, tenho que fazer xixi até sentado (…) para a urina solta não tem remédio (E02, E06, E15, E34, E38 e E42)*.

*DSC H – Mudanças, sequelas variadas e consequências negativas*, 23,33% (n = 14)

*A retirada da próstata para um homem como eu, que sempre conviveu com ela, não é por causa de machismo, mas faz muita falta. Você se sente como um homem menosprezado, porque de homem você não tem ação (…) depois que eu operei não sou ninguém mais, o fato de usar fraldas acabou comigo. Se pudesse fazer um implante de próstata ia ser muito bom para os homens, porque quando tira a próstata arrebenta com o homem (…) A cirurgia me salvou a vida, mas eu não estou vivendo (E03, E11, E12, E14, E17, E30, E38, E39, E41, E46, E49, E55, E58 e E60)*.

### 5) Desafios biopsicossociais: sofrimento, preocupação e choque (DSC G e DSC J)

*DSC G – Sofrimento, experiência ruim demais, difícil, desagradável e triste*, 15% (n = 9)

*A cirurgia em si é desagradável, é frustrante. A gente passa por momentos difíceis. Passamos mal, porque corta a gente e sentimos muita dor até uns cinco dias. Pra mim só foi sofrimento, não tenho nada de bom pra falar (…) é triste, tive que passar pelo psicólogo, porque o nome câncer já assusta. Eu acho que deveria ter um preparo psicológico maior (E12, E19, E20, E30, E34, E39, E45, E51 e E55)*.

*DSC J – Susto, preocupação, choque*, 6,67% (n = 4)

*Fiquei bem preocupado, porque todo mundo fala, todos os homens falam, que a retirada da próstata acaba com a tua vida sexual! Foi um choque muito grande, no começo é um susto (…) custei a voltar no lugar, fiquei meio sem chão (E19, E29, E30 e E39)*.

A Figura 2 representa o painel dos discursos, de acordo com os agrupamentos.

**Figura 2:** Ilustração síntese das representações sociais sobre a cirurgia de retirada da próstata a partir das significações dos homens pelo discurso do sujeito coletivo, Alfenas, MG, Brasil, 2025.

## DISCUSSÃO

Os homens perceberam que a cirurgia proporcionou o fim do sofrimento e dos sintomas que eles classificaram como ruins, conforme o DSC A. A percepção de melhora dos sintomas urinários e também da dor óssea foi evidenciada em estudo realizado com participantes em estado avançado de câncer de próstata após a cirurgia de orquiectomia, demonstrando que os procedimentos cirúrgicos são reconhecidos como proporcionadores de bem estar^(13)^. No entanto, a melhora nos sintomas do trato urinário inferior após a prostatectomia não ocorre imediatamente, pois pode levar de seis a 12 meses, o que aponta para a importância do suporte profissional para o autogerenciamento desses sintomas até que se estabilizem^(14)^.

A percepção positiva da cirurgia também foi atribuída ao fato de que a cirurgia remove um problema que os homens consideravam ter, mas que não podiam ver, ou seja, ^removendo-^ se o câncer, também removem-se as preocupações. A literatura aponta que homens em vigilância ativa manifestaram ansiedade em relação à progressão do câncer, por essa modalidade terapêutica não ser definitiva^(15)^. Dessa maneira, compreende-se que o tratamento definitivo, como a remoção cirúrgica, pode simbolizar um controle mais adequado do câncer para alguns homens.

No DSC E, a cirurgia foi vista como um procedimento que removeu uma parte do corpo que estava com problemas, que não estava funcionando bem, e que poderia causar a morte, demonstrando uma familiarização com o câncer e suas consequências por meio da ancoragem. Resultado de estudo corrobora esse discurso, uma vez que os homens, temendo a progressão da doença, viram na opção cirúrgica a única maneira de remover esse medo, curar o câncer e prolongar a vida^(16)^.

Ademais, a cirurgia foi vista como uma benção divina, por acabar com o sofrimento, trazer a cura e evitar a morte. Em consonância com estes resultados, aqueles que optaram pela remoção cirúrgica da próstata relataram alívio por livrarem seus corpos do câncer e que Deus os havia abençoado^(15)^.

No DCS F, a prostatectomia foi vista como uma intervenção necessária frente um tumor maligno, que poderia, de forma lenta, se agravar e se espalhar. Para representar a decisão pela prostatectomia, utilizaram termos técnicos, como “tumor maligno”, ao mesmo tempo que ancoraram sentidos ao descreverem as consequências do câncer. Tal resultado coaduna com os achados de estudo, no qual os participantes reconheceram a necessidade do procedimento cirúrgico, mesmo que isso significasse perdas relacionadas às funções sexuais e urinárias, e que diante desse dilema, a opção pela vida foi maior do que as consequências negativas advindas da cirurgia^(16)^.

No DSC K, os participantes alegaram o desconhecimento da importância da prostatectomia e arrependeram-se de não terem sido submetidos a ela há mais tempo. Estudo que avaliou experiências de homens e o impacto no seu bem-estar demonstrou que aqueles que realizaram a radioterapia e tiveram recorrência do câncer de próstata acreditaram que a remoção total da próstata teria evitado esse problema^(17)^.

Esse DSC ratifica a necessidade de os profissionais de saúde oferecerem esclarecimentos sobre as opções terapêuticas, seus riscos e benefícios de maneira personalizada^(18)^, respeitando-se os princípios da literacia em saúde, com linguagem que facilite a compreensão, promovendo a autonomia no processo decisório quanto à terapêutica, pois a ocorrência de efeitos colaterais e a recorrência do câncer podem levá-los a dúvidas quanto à eficácia da opção escolhida e à confiança nos profissionais de saúde^(18,19)^.

No DSC C, os homens apresentaram relatos de que a prostatectomia não causou sequelas e nem mudanças em suas vidas. A eficácia do tratamento, a ausência de dor, a preservação da função sexual e urinária podem se relacionar com essas representações, uma vez que, como dizem, essas são consequências que podem afetar outros de seu meio de pertença.

Percepções similares foram encontradas em estudo, em que o participante referiu que se sentia com muita sorte, pois mesmo apresentando disfunção erétil, ele, por meio de recursos medicamentosos, conseguia manter as relações sexuais, ao contrário de outros homens que ele conhecia^(15)^. Em outra pesquisa, a autopercepção “alguém que teve câncer”, associada ao pouco comprometimento no bem-estar geral, foi assumida por homens mais jovens e sexualmente mais ativos^(5)^. Assim, compreende-se que manter a função sexual, mesmo que com auxílio da terapêutica, faz com que os homens representem a cirurgia como algo que pouco ou nada impactou em suas vidas.

No DSC I, para os participantes, não adianta lamentar a realização da prostatectomia, pois é necessário cuidar da próstata quando se é mais novo, evitando assim, o câncer como consequência. Resultado de pesquisa constatou que os participantes referiram que hábitos nocivos, como o consumo de álcool e tabaco, causaram o câncer, e na juventude não se pensa nas consequências desses hábitos^(20)^.

Em outro estudo, os homens após 10 anos de prostatectomia relataram que o melhor conselho aos outros homens é sobre a importância dos cuidados de saúde. Essa experiência, de acordo com os autores, foi uma maneira de os homens se conectarem com a masculinidade coletiva^(15,21)^. Diante disso, as experiências com o câncer de próstata e com a cirurgia fizeram com que os homens valorizassem os cuidados relacionados às medidas para a prevenção da doença.

Para os participantes do presente estudo, realizar a prostatectomia pode ser motivo para as pessoas “zombarem” pelas sequelas decorrentes da cirurgia; no entanto, eles não se sentiram envergonhados diante dessa realidade. O estigma do câncer de próstata que permeia o universo simbólico de alguns homens pode levar à vulnerabilidade, fazendo com que os homens que se submeteram a prostatectomia escondam suas lutas e mantenham segredo sobre os efeitos adversos da cirurgia, culminando em isolamento social^(22)^.

Dito isso, entende-se que os homens precisam vencer o constrangimento que permeia as crenças sobre a masculinidade, para que, assim, possam obter o apoio de que necessitam para o enfrentamento do câncer e das sequelas advindas dos procedimentos terapêuticos.

O peso da representação da perda da função sexual, evidenciado no DSC B, levou os homens a questionarem a relevância dos esclarecimentos pelos profissionais de saúde, o que poderia orientá-los à não realização da cirurgia. Resultados similares foram encontrados em artigo, em que os homens se arrependeram da realização da prostatectomia, após o conhecimento obtido por meio de leituras de textos científicos, pois entenderam que adiando o procedimento poderiam ter mais tempo de vida sexual^(15)^. Reconhece-se, nesse discurso, o peso que a perda da função sexual representa, uma vez que o conhecimento desse problema poderia orientá-los a não realizar a cirurgia.

Para os participantes, a prostatectomia foi percebida como uma “mutilação”, representação que ancora a remoção de uma parte do corpo e de sua função. Resultados de pesquisa apontaram que a alteração da função erétil fez com que os homens se sentissem incapazes de cumprir seu dever como maridos no relacionamento sexual do casal^(16)^. Dito isso, as esposas devem ser inseridas no cuidado, para que assim possam discutir, junto com cônjuge e profissionais de saúde, os efeitos adversos da prostatectomia^(23)^.

Diante dessa realidade, tem-se defendido a importância da pré-reabilitação, que consiste em uma abordagem multiprofissional antes do procedimento cirúrgico, que na prostatectomia consiste em abordar potenciais efeitos colaterais, antes que eles se manifestem após a cirurgia. Essa abordagem objetiva a melhoria da capacidade funcional do homem, a resiliência psicológica e a saúde geral, aliviando o impacto negativo da cirurgia na qualidade de vida. Dentre as intervenções utilizadas nesta fase estão: atividade física, apoio de pares (por exemplo: grupo de homens com câncer), treinamento dos músculos do assoalho pélvico, orientações nutricionais e administração de inibidores da fosfodiesterase-5^(24)^. Essa abordagem é fundamental para que os homens possam reelaborar melhor os efeitos adversos advindos da prostatectomia, motivando-os a buscar recursos para mitigar esses efeitos.

Quanto ao DSC D, os participantes disseram que após a cirurgia perceberam a perda do controle urinário, exigindo mudanças de hábitos, como a posição para usar o vaso sanitário e o uso de fraldas. Neste discurso, os participantes se apropriam da imagem de um chuveiro para ancorar representações referentes a maneira como entendem o atual padrão urinário.

Resultados similares foram encontrados em estudos em que os homens relataram que a incontinência afeta o sono, pois as fraldas e os absorventes não são totalmente eficientes para controlar o volume urinário durante a noite, o que o atrapalha a dormir^(15)^, além de fazer com que os homens evitem ocasiões sociais^(25)^, sintam-se constrangidos ao usarem banheiros públicos^(26)^, tenham problemas em realizar viagens, preocupem-se em estar com mau odor e restrinjam o consumo de líquidos^(25,27)^. E ainda, o uso de fraldas foi apontado como algo que os despersonificou, fazendo com que os homens se sintam como crianças^(14)^.

Em vista desse contexto, entendem-se os impactos que a perda do controle esfincteriano pode representar, alterando o modo como os homens vivem, comportam-se e estabelecem suas relações sociais.

Nesse âmbito, como estratégia para o acompanhamento dos homens com incontinência, pesquisadores brasileiros desenvolveram o aplicativo IUProst®, sendo considerado uma tecnologia que pode favorecer os cuidados prestados pelos enfermeiros, uma vez que esse aplicativo oferece informações sobre mudanças nos hábitos de vida e realização de exercícios para fortalecimento dos músculos pélvicos, permitindo a realização dos exercícios de forma remota, por meio de um smartphone^(28)^.

No DSC H, o implante de próstata foi representado como uma alternativa em substituição à próstata removida. Infere-se que a próstata é representada como um órgão associado à capacidade erétil, de modo que um implante os faria retomar essa função.

Para o sujeito coletivo, o procedimento cirúrgico foi representado como algo para salvar a vida, no entanto, o impacto negativo das sequelas modificou a percepção, demonstrando que o sentido de viver está atrelado não somente a não morrer, mas em como se vive.

Nesse contexto, a enfermagem deve buscar abordagens inovadoras de cuidado, que contribuam para a melhoria da qualidade de vida. Evidências de cuidado colaborativo demonstram que as habilidades profissionais de enfermagem, combinadas com a pesquisa científica, com foco nas necessidades individuais, foram eficazes para o desenvolvimento de planos de cuidados personalizados para homens após a prostatectomia, uma vez que bem-estar emocional, melhor capacidade de autocuidado e qualidade de vida e redução de complicações pós-operatórias foram evidenciados^(29)^.

No DSC G, a cirurgia foi percebida como algo desagradável e frustrante, que o fez passar por momentos difíceis, causando mal-estar físico e psicológico, decorrentes do procedimento e também da palavra câncer. Estudo demonstra que a representação social do câncer para pessoas adultas em tratamento oncológico, em um primeiro momento, permeia sentidos relacionados à doença, à tristeza e à morte, em que a tristeza se apresenta como um sentimento negativo diante da possibilidade do adoecimento e a morte como uma relação direta atrelada ao diagnóstico de câncer^(7)^. Nessas circunstâncias, há a necessidade do diálogo e do acompanhamento psicológico, para que assim, os homens possam enfrentar o tratamento com mais tranquilidade.

Resultado de estudo corrobora esses achados, tendo em vista que os homens em tratamento para o câncer de próstata lidam com problemas psicológicos contínuos relacionados ao câncer e ao seu tratamento, o que gerou impactos na autoestima e nos relacionamentos. Apontaram ainda que a busca por apoio para seus problemas psicossociais é algo desafiador, pois existe constrangimento na discussão dessas questões^(30)^.

No DSC J, as representações que permeiam o universo consensual masculino referiram preocupação pela perda da função sexual, como observado na literatura, em que as preocupações em torno dos possíveis resultados indesejados fizeram com que os homens apresentassem incertezas sobre qual tratamento deveriam optar, pois, ao mesmo tempo que percebiam melhores resultados por meio da cirurgia, apresentavam incertezas em relação aos riscos de disfunção urinária e sexual, sendo esse o fator central no processo decisório^(17)^.

Assim, apreende-se que as representações sobre os efeitos indesejados da prostatectomia permeiam o universo consensual masculino, causando preocupações, o que pode nortear o processo decisório terapêutico.

Ainda, relataram que foi um choque e um susto. Infere que os homens representaram a cirurgia como algo que abala, que os tira do lugar e dificilmente voltam ao “normal”. Esse lugar pode simbolizar sua identidade enquanto homens saudáveis, com controle de seus corpos e funções. Reitera-se a importância de que os homens sejam esclarecidos sobre o procedimento cirúrgico, tendo em vista que a maioria dos participantes sentiu que não tinha as informações necessárias para gerenciar os efeitos colaterais de longo prazo, e que não sabiam como voltariam ao “normal”, de modo que desconheciam os recursos disponíveis para gerenciar seus problemas contínuos^(30)^.

Como limitações desse estudo, considera-se que não foi possível identificar situações de remissão da doença, o tipo de abordagem cirúrgica laparoscópica ou aberta e preservação de feixes neuro vasculares, bem como dados relacionados à incontinência urinária e disfunção sexual, o que poderia contribuir para uma melhor caracterização dos participantes. Ademais, as entrevistas não retornaram aos participantes, devido ao fluxo de atendimento do centro oncológico.

Como contribuições para a prática, os resultados revelaram a importância da literacia em saúde para a compreensão das orientações, o que contribui para a coparticipação do homem nas decisões terapêuticas, e ainda, a participação das companheiras/esposas nas orientações sobre a prostatectomia e seus efeitos adversos. Para a efetividade dessas orientações, devem-se criar espaços de dialogicidade que permitam aos homens vencer as barreiras do constrangimento e obter o apoio que necessitam.

As estratégias personalizadas, inovadoras, embasadas no autocuidado apoiado e em evidências científicas atuais podem contribuir para amenizar o sofrimento, melhorar as funções físicas relacionadas à sexualidade e à função urinária, além da compreensão da doença e dos tratamentos.

## CONSIDERAÇÕES FINAIS

A análise das representações sociais revelou que, para alguns homens, a prostatectomia radical foi vista como um procedimento benéfico e curativo, um cuidado de saúde necessário, demonstrando os benefícios e a satisfação com o tratamento. Por outro lado, outros discursos expressaram a prostatectomia como uma experiência relacionada ao sofrimento, aos prejuízos na função sexual, na virilidade, no controle urinário e na relação conjugal. A ausência da próstata foi lastimada, em que sentem que cirurgia salvou a vida, mas que não estão vivendo.

Sugere-se a realização de estudos em outros contextos socioculturais e a demarcação do tempo de pós-operatório, bem como das fases de tratamento e de acompanhamento.

## Data Availability

Todo o conjunto de dados que dá suporte aos resultados deste estudo foi disponibilizado no SciELO Data e pode ser acessado em https://doi.org/10.48331/SCIELODATA.AJLTDE.

# Translation: Representaciones sociales de la prostatectomía radical desde la perspectiva de los hombres sometidos a la cirugía^*^

**DOI:** 10.1590/1980-220X-REEUSP-2025-0127es

**Authors:** Bianca de Moura Peloso-Carvalho, Rogério Silva Lima, José Vitor da Silva, Eliza Maria Rezende Dázio, Murilo César do Nascimento, Silvana Maria Coelho Leite Fava

**Affiliations:** 1 Universidade Federal de Alfenas, Programa de Pós-graduação em Enfermagem, Alfenas, MG, Brasil.; 2 Universidade Federal de São Carlos, Departamento de Gerontologia, São Carlos, SP, Brasil.

**Keywords:** Prostatectomia, Neoplasias de la Próstata, Investigación Cualitativa, Representación Social, Enfermería Oncológica

## Abstract

**Objetivo:**

Comprender las representaciones sociales de la prostatectomía radical desde la perspectiva de los hombres sometidos a la cirugía.

**Método:**

Investigación cualitativa, descriptiva, basada en la teoría de las Representaciones Sociales. Participaron 60 hombres, diagnosticados con cáncer de próstata, sometidos a prostatectomía radical, atendidos en un municipio del sur de Minas Gerais. Recolección de datos entre febrero y septiembre de 2022, mediante entrevistas y acceso a los expedientes clínicos. Los datos de caracterización se tabularon y presentaron en frecuencia absoluta y relativa, y los cualitativos se transcribieron y analizaron mediante el método del Discurso del Sujeto Colectivo.

**Resultados:**

Se identificaron 11 Ideas Centrales, asociadas a los impactos negativos en las funciones sexuales, urinarias y en la identidad masculina, a la neutralidad, mediante la adaptación satisfactoria y a las percepciones positivas: curación, alivio y satisfacción, además de la decisión y la confianza en el procedimiento quirúrgico.

**Consideraciones Finales:**

Las representaciones sociales revelaron una complejidad de experiencias relacionadas con la satisfacción, la adaptación y la insatisfacción con los resultados posquirúrgicos.

## INTRODUCCIÓN

El cáncer de próstata es la neoplasia más común en hombres en 112 países, lo que representa el 15% de los tipos de cáncer. Los cambios demográficos mundiales y el aumento de la esperanza de vida sugieren que el número de casos nuevos por año aumentará de 1,4 millones en 2020 a 2,9 millones en 2040^(1)^. A nivel nacional, se configura como el procedimiento de mayor incidencia, sin considerar los tumores de piel no melanoma^(2)^.

En cuanto a la terapia, la prostatectomía radical es el procedimiento más adecuado para el tratamiento del cáncer de próstata clínicamente localizado, considerando el control de la enfermedad y la mortalidad por cáncer. Este procedimiento consiste en la resección total de la próstata, las vesículas seminales, los ganglios linfáticos y otras estructuras pélvicas afectadas, y puede realizarse por vía abierta, laparoscópica y robótica^(3)^. En cuanto al número de cirugías realizadas al año, la Sociedad Brasileña de Urología ha proyectado para 2025 la realización de 21.219 procedimientos quirúrgicos para el tratamiento del cáncer de próstata^(4)^.

Este procedimiento quirúrgico puede presentar, como consecuencias no deseadas, incontinencia urinaria y disfunción eréctil, que son efectos adversos que contribuyen al impacto de la cirugía en la calidad de vida^(5)^, de modo que las representaciones atribuidas a la salud, la masculinidad, las pérdidas funcionales y la autopercepción influyen en la forma en que los hombres entienden y experimentan estos impactos, lo que culmina en una mejor o peor forma de afrontar la enfermedad, lo que exige la comprensión por parte de los profesionales de la salud^(6,7)^.

El marco de referencia de la Teoría de las Representaciones Sociales ofrece un marco teórico que permite captar aspectos simbólicos y construidos colectivamente, a través de la aproximación al universo consensuado y reificado. En este sentido, este marco permite comprender cómo los hombres entienden y afrontan su enfermedad, considerando que los fenómenos se procesan como eventos singulares y subjetivos, al mismo tiempo que son socialmente elaborados y compartidos^(7,8)^.

Los estudios cualitativos que buscan explorar la experiencia masculina en relación con la prostatectomía a la luz de la teoría de las Representaciones Sociales aún son incipientes en el ámbito nacional e internacional, dado el contexto epidemiológico del cáncer de próstata y el número de procedimientos quirúrgicos realizados. Ante lo expuesto, este estudio tiene como objetivo comprender las representaciones sociales de la prostatectomía radical desde la perspectiva de los hombres sometidos a la cirugía.

La enfermería, como área central en el cuidado del hombre que se somete a una prostatectomía, puede beneficiarse directamente de los hallazgos de esta investigación, en la medida en que las repercusiones subjetivas de la cirugía, más allá de los aspectos clínicos, pueden subsidiar la implementación de intervenciones asistenciales y educativas que consideren aspectos emocionales, relacionales e identitarios del postoperatorio, con el fin de contribuir al avance de la ciencia de la enfermería en este escenario de cuidados e investigación.

## MÉTODO

### Tipo de Estudio

Se trata de una investigación descriptiva, de enfoque cualitativo, basada en el marco teórico de la Teoría de las Representaciones Sociales y el marco metodológico del Discurso del Sujeto Colectivo (DSC)^(7,8)^.

Las representaciones sociales, como sentido común, están presentes en las opiniones, los discursos, las posturas, los mensajes y las imágenes de los medios de comunicación, y se consideran formas de comprensión y comunicación, situándose entre las percepciones captadas por la vida cotidiana y los significados atribuidos por las personas^(8)^.

La técnica del DSC permite acceder a las Representaciones Sociales, en las que las expresiones individuales se agrupan en categorías que contienen opiniones, experiencias y vivencias individuales y colectivas. Para construir el DSC, el investigador debe trabajar con las siguientes figuras metodológicas: las expresiones clave, que son fragmentos continuos o discontinuos del discurso, que corresponden a las respuestas relativas a la pregunta orientadora; las Ideas Centrales, que son las expresiones que describen de manera más sintética el sentido o los sentidos de las Expresiones Clave; y el DSC, que es la unión de las Expresiones Clave relacionadas con las Ideas Centrales que tienen el mismo sentido. Estas expresiones deben reunirse, editarse y redactarse en primera persona del singular para configurar el DSC^(9)^.

La realización de este estudio y la elaboración de su informe cumplieron con los principios de los Criterios Consolidados para la Presentación de Informes de Investigación Cualitativa (COREQ)^(10)^.

### Lugar del Estudio

La investigación se llevó a cabo en un centro oncológico de alta complejidad, que atiende a 26 municipios del sur de Minas Gerais. La institución consta de seis plantas, la primera de las cuales está destinada a consultas multiprofesionales y radioterapia, la segunda a quimioterapia y hormonoterapia y las demás a hospitalización, con 84 camas equipadas. Cuenta con un equipo compuesto por médicos, enfermeros, farmacéuticos, psicólogos, trabajadores sociales y nutricionistas que trabajan de forma integrada.

### Población del Estudio y Criterios de Selección

Se adoptaron como criterios de inclusión los hombres diagnosticados con cáncer de próstata (CID C61), mayores de 18 años, que estaban en tratamiento o seguimiento oncológico, que se sometieron a una cirugía de prostatectomía radical, independientemente de la técnica utilizada en el procedimiento y del momento en que se realizó. Los criterios de exclusión fueron los hombres que presentaban alguna dificultad para comprender la investigación y/o participar en el estudio y que, mediante la aplicación del Cuestionario de Evaluación Mental^(9)^, acertaban menos de siete de las diez preguntas del instrumento.

Se contactó a 71 hombres. De ellos, siete se negaron a participar en el estudio, uno fue excluido debido a dificultades para comprender la investigación y tres porque, aunque en sus expedientes clínicos constaba que se habían sometido a una prostatectomía, negaron haberse sometido al procedimiento. De este modo, 60 hombres formaron parte de este estudio, constituyendo una muestra por conveniencia.

### Recolección de Datos

El periodo destinado a la recolección de datos abarcó los meses de febrero a septiembre de 2022, en los que, bajo la supervisión de las secretarias de la consulta, la primera autora del trabajo, maestra en enfermería con experiencia en la recolección de datos cualitativos, accedió a los expedientes clínicos de los posibles participantes, que esperaban en la sala de espera de la consulta de oncología, para realizar el estudio de los hombres sometidos a cirugía de prostatectomía.

Una vez en posesión de estos datos, la investigadora se dirigía cordialmente a los hombres en la sala de espera y los invitaba a acompañarla a una sala privada cercana a los consultorios, con el fin de garantizar un entorno seguro y evitar interrupciones y ruidos. Si los hombres venían acompañados, se invitaba a sus acompañantes a participar solo en este momento inicial, es decir, la invitación a participar en el estudio y las aclaraciones sobre los objetivos y los procedimientos de la investigación. Tras el consentimiento del participante, se invitaba a los acompañantes a esperar en la sala de espera hasta que finalizara la recolección de datos. En la sala privada solo permanecían la investigadora y el participante, siendo este el primer contacto. Se aplicaba entonces, el Cuestionario de Evaluación Mental^(11)^ y, en función del resultado, se continuaba con la recolección mediante la realización de la entrevista.

El formulario de la entrevista estaba compuesto por preguntas relacionadas con las características sociodemográficas y clínicas de los participantes y preguntas orientativas. En cuanto a las características, las preguntas se referían a la edad, el estado civil, con quién vive, el nivel de estudios, los ingresos mensuales aproximados, la ocupación, el tratamiento realizado, el año en que se realizó la cirugía de prostatectomía y la pregunta orientadora: Cuénteme, por favor, ¿qué significa para usted la cirugía de extirpación de la próstata?

Cabe destacar que las entrevistas se realizaron únicamente sobre la base de la pregunta orientadora, de acuerdo con el método DSC^(9)^.

Se realizó una entrevista con cada participante, y la transcripción de los datos no se les devolvió. La logística y la dinámica del servicio no permiten el cumplimiento de este punto previsto por el COREQ, ya que cada participante presenta una dinámica de tratamiento y el consiguiente regreso al consultorio.

Los testimonios se grabaron en audio y se registraron en dos teléfonos celulares, un Xiaomi^R^ y un Samsung^R^, con una aplicación de grabadora, de manera simultánea. Las entrevistas tuvieron una duración media de aproximadamente 30 minutos. La recolección de datos en los expedientes clínicos se llevó a cabo con la ayuda del equipo del sector de archivos, que organizó los registros de los participantes. El procedimiento fue supervisado por los profesionales del sector y se destinó a la obtención de datos relativos a la fecha de realización de la cirugía y a los tratamientos realizados.

La recolección de datos se llevó a cabo durante el periodo de pandemia de COVID-19, por lo que se adoptaron las siguientes medidas de bioseguridad: los lugares de las entrevistas estaban ventilados, de modo que tanto los participantes como la investigadora utilizaron cubrebocas y mantuvieron una distancia física de 1,5 m; además, ambos se desinfectaron las manos con gel de alcohol al inicio y al final de cada entrevista.

### Análisis y Tratamiento de los Datos

Los datos correspondientes a la caracterización sociodemográfica y clínica se tabularon en el programa Microsoft Excel^R^2010 y se presentaron en formato de texto, utilizando distribuciones absolutas y relativas. Los datos cualitativos se transcribieron íntegramente con la ayuda del editor de texto Microsoft Word 2010, respetando la oralidad.

Los datos cualitativos se exploraron mediante una lectura vertical y horizontal extensa y rigurosa de los discursos individuales, optando por utilizar el Instrumento de Análisis del Discurso 1-IAD1 y el Instrumento de Análisis del Discurso 2-IAD2, que permiten una organización sistemática de las expresiones clave y las ideas centrales, con el fin de posibilitar la fidelidad interpretativa de los discursos, ampliando la transparencia en el proceso analítico^(12)^.

Tras el análisis de los datos textuales y las expresiones clave, se agruparon las ideas centrales iguales, similares y complementarias. A continuación, se identificaron los significados emergentes y los participantes que contribuyeron a cada representación; y, por último, se elaboraron los Discursos del Sujeto Colectivo, en los que tres investigadores con dominio del método participaron en esta etapa de análisis, garantizando el rigor analítico y la fidelidad a las narrativas.

### Aspectos Éticos

La investigación se inició tras la aprobación del proyecto por el Comité de Ética en Investigación de la Universidad Federal de Alfenas, con el número de dictamen y CAAE: 5.131.466.

Tras comprender el objetivo del estudio y aceptarlo, los participantes recibieron el Término de Consentimiento Libre e Informado, que contenía información clara y concisa sobre la investigación, los objetivos, los procedimientos, los posibles beneficios y riesgos, además de los aspectos éticos aplicados a la investigación con seres humanos, de acuerdo con la Resolución del Consejo Nacional de Salud (CNS) 466/12.

Tras firmar su aceptación de participar, se entregó una copia del formulario al participante y otra quedó en poder de la investigadora. Se preservó la identidad de los participantes y sus nombres personales fueron sustituidos por códigos con la inicial E de entrevistado, seguida del número en el orden en que fueron abordados por la investigadora, como: E1, E2, E3, sucesivamente.

## RESULTADOS

De los 60 hombres que componían la población del estudio, en lo que respecta a la edad, el intervalo era de 51 a 87 años, la media era de 68,7 años y el 41,67% (n = 25) tenían entre 60 y 69 años y el 40% (n = 24) entre 70 y 79 años. Se observó un predominio entre aquellos que declararon vivir solo con su esposa, 51.67% (n = 31), y haber completado la educación primaria, 36.67% (n = 22).

En cuanto al estado civil, los ingresos y la ocupación, estas características se analizaron antes y después de la cirugía de prostatectomía. En cuanto al estado civil antes de la cirugía, la mayoría de los hombres estaban casados o en unión libre, 90% (n = 54). Después de la cirugía, solo dos hombres casados quedaron viudos, mientras que los demás permanecieron en la misma situación.

En cuanto al ingreso mensual aproximado, 36,67% (n = 22) declararon recibir mensualmente un salario mínimo. Después de la prostatectomía, el 80% (n = 48) no observó cambios en sus ingresos, mientras que el 13,33% (n = 8) informó un empeoramiento y el 6,67% (n = 4) indicó una mejora.

En cuanto a la ocupación, el 61,67% (n = 37) estaba jubilado antes de la cirugía y permaneció en esa condición. Entre los que trabajaban, el 38.33% (n = 23), el 5% (n = 3) se jubiló después de la cirugía, el 1.67% (n = 1) quedó desempleado y el 1.67% (n = 1) cambió de profesión, pasando de albañil a agricultor.

En cuanto a la caracterización clínica, las cirugías se realizaron entre 2005 y 2022, con la distribución más significativa, 46.67% (n = 28), en los últimos cuatro años, según la Figura 1.

**Figura 1:** Distribución temporal de los años en que los participantes del estudio se sometieron a una prostatectomía. Alfenas, MG, 2025 (n = 60).

A partir del análisis de los datos, se identificaron 11 discursos del sujeto colectivo, que se agruparon en cinco temas de discusión, según significados similares. A continuación, se presentan fragmentos de los discursos, indicando la frecuencia relativa al número de participantes que contribuyeron con cada discurso, así como la identificación codificada de dichos participantes.

### 1) Percepciones positivas: curación, alivio y satisfacción con la cirugía (DSC A y DSC E)

*DSC A – Fue bueno, una maravilla, 36,67%* (n = 22)

*Mira, para mí fue muy bueno, realmente genial, porque ya no sufrí más, acabó con todo lo malo que estaba sintiendo (…) sentía mucho dolor al orinar y también me sentía cansado sin motivo, desanimado (…) La cirugía te quita eso de la cabeza, ese problema que tenemos, pero no podemos ver (E01, E04, E06, E12, E15, E16, E17, E22, E23, E25, E29, E33, E35, E40, E47, E48, E50, E51, E53, E54, E56 y E60)*.

*DSC E – Eliminar el problema, no morir, curar el cáncer*, 38,33% (n = 23)

*Significa que eliminó la parte que tenía el problema. Si no me hubieran extirpado la próstata, hoy estaría bajo tierra (…) creo que curó el cáncer. Siento que he renacido (…) Es algo que Dios nos ha dado para curarnos y no morir antes, es una bendición. Fue un alivio para mí, empecé a sentirme mucho mejor (E04, E06, E10, E13, E14, E15, E17, E19, E21, E22, E26, E37, E38, E39, E45, E47, E48, E50, E52, E54, E56, E57 y E60)*.

### 2) Decisión y confianza: procedimiento quirúrgico necesario (DSC F y DSC K)

*DSC F – Era algo que había que hacer, si no, el tumor crecería y empeoraría*, 41,67% (n = 25)

*Mira, fue una intervención necesaria debido a un tumor maligno, un cáncer. Decidí operarme (…) porque si lo dejaba, aumentaría y luego empeoraría, poco a poco se va apoderando de uno (…) significa cuidar la salud (…) el médico ME dijo: “Si te operas, es más seguro” (E04, E06, E10, E13, E14, E15, E17, E19, E21, E22, E26, E27, E36, E37, E38, E39, E45, E47, E48, E50, E52, E54, E56, E57 y E60)*.

*DSC K – Algo que ya se podría haber hecho,* 3,33% (n = 2)

*Podría haberme operado hace mucho tiempo, pero pensaba que no era tan importante, no lo sabía. Sufrí durante unos cinco o seis años tomando medicamentos, podría haberme aliviado antes, pero aun así fue bueno (E22 y E56)*.

### 3) Normalización: adaptación y conformidad (DSC C y DSC I)

DSC C – No ha cambiado nada, vida normal, 28,33% (n = 17)

*En cuanto a la cirugía, no me perjudicó en nada, mi vida sigue siendo normal (…) no sentí dolor, no sentí nada. (…) Mi función sexual sigue funcionando (…), pero sé que para muchos hombres no es así. Además, tuve suerte de no tener problemas con la orina (…) Y hay personas que necesitan quimioterapia, radioterapia, y yo no (…) (E01, E02, E04, E07, E10, E18, E20, E21, E31, E41, E43, E44, E45, E48, E50, E51 y E53)*.

*DSC I – Es algo a lo que uno se acostumbra, no sirve de nada lamentarse*, 6,67% (n = 4)

*Mira, ya han pasado dos años desde que me operaron y hoy estoy un poco más acostumbrado a la situación (…) hay que cuidar la próstata cuando se es más joven, porque después de que pasan las cosas, no sirve de nada lamentarse (…) no me da vergüenza ni me avergüenza hablar de ello con nadie. Mucha gente puede burlarse, pero a mí no me importa. Lo que importa es mi salud (…) y tengo a mi esposa a mi lado (E12, E18, E30 y E52)*.

### 4) Impactos negativos: función sexual, función urinaria e identidad masculina (DSC B, DSC D y DSC H);

*DSC B – Compromiso en la función sexual*, 40% (n = 24)

*Si hubiera sabido que iba a quedar así, no me habría operado (…) porque la cirugía tiene un efecto perjudicial, que es en la relación sexual (…) Creo que los médicos deberían explicarnos el postoperatorio (…) Siento que me falta algo, es como si estuviera mutilado (…) La cirugía elimina el placer y gran parte de lo que el hombre ejerce como ser humano, como macho, su virilidad se ve comprometida. Ya no tengo relaciones con mi esposa, no valgo nada y eso es desagradable (E01, E02, E03, E04, E08, E09, E15, E16, E19, E23, E24, E26, E29, E30, E31, E32, E34, E36, E39, E51, E52, E58, E59 y E60)*.

*DSC D – Problemas urinarios*, 10% (n = 6)

*(…) Después de la operación (…) mi orina se vuelve fluida, uso pañales desechables todo el tiempo. Si me quedo quieto, mi orina se controla, pero si empiezo a toser o a hacer fuerza, la orina se escapa poco a poco. Esto ocurre porque me pusieron una sonda, que permanece durante 15 días y dilata. Hoy en día, mi orina parece una ducha, no hay inodoro que la aguante, tengo que orinar incluso sentado (…) para la incontinencia urinaria no hay remedio (E02, E06, E15, E34, E38 y E42)*.

*DSC H – Cambios, secuelas variadas y consecuencias negativas*, 23,33% (n = 14)

*La extirpación de la próstata para un hombre como yo, que siempre ha convivido con ella, no es por machismo, pero se echa mucho de menos. Te sientes como un hombre menospreciado, porque como hombre no tienes acción (…) después de la operación ya no soy nadie, el hecho de usar pañales me ha destrozado. Si se pudiera hacer un implante de próstata sería muy bueno para los hombres, porque cuando te quitan la próstata te destrozan como hombre (…) La cirugía me salvó la vida, pero no estoy viviendo (E03, E11, E12, E14, E17, E30, E38, E39, E41, E46, E49, E55, E58 y E60)*.

### 5) Desafíos biopsicosociales: sufrimiento, preocupación y conmoción (DSC G y DSC J)

*DSC G – Sufrimiento, experiencia muy mala, difícil, desagradable y triste*, 15% (n = 9)

*La cirugía en sí es desagradable, es frustrante. Pasamos por momentos difíciles. Nos sentimos mal, porque nos cortan y sentimos mucho dolor hasta unos cinco días. Para mí solo fue sufrimiento, no tengo nada bueno que decir (…) es triste, tuve que acudir al psicólogo, porque la palabra cáncer ya da miedo. Creo que debería haber una mayor preparación psicológica (E12, E19, E20, E30, E34, E39, E45, E51 y E55)*.

*DSC J – Susto, preocupación, conmoción*, 6,67% (n = 4)

*Me preocupé mucho, porque todo el mundo dice, todos los hombres dicen, que la extirpación de la próstata acaba con tu vida sexual. Fue un shock muy grande, al principio es un susto (…) me costó recuperarme, me quedé un poco desorientado (E19, E29, E30 y E39)*.

La Figura 2 representa el panel de los discursos, según los grupos.

**Figura 2:** Ilustración síntesis de las representaciones sociales sobre la cirugía de extirpación de la próstata a partir de los significados de los hombres por el discurso del sujeto colectivo, Alfenas, MG, Brasil, 2025.

## DISCUSIÓN

Los hombres percibieron que la cirugía proporcionó el fin del sufrimiento y de los síntomas que ellos clasificaron como malos, según el DSC A. la percepción de mejora de los síntomas urinarios y también del dolor óseo se evidenció en un estudio realizado con participantes en estado avanzado de cáncer de próstata después de la cirugía de orquiectomía, lo que demuestra que los procedimientos quirúrgicos se reconocen como proveedores de bienestar^(13)^. Sin embargo, la mejora de los síntomas del tracto urinario inferior tras la prostatectomía no se produce de forma inmediata, ya que puede tardar entre seis y doce meses, lo que apunta a la importancia del apoyo profesional para el autocontrol de estos síntomas hasta que se estabilicen^(14)^.

La percepción positiva de la cirugía también se atribuyó al hecho de que la cirugía elimina un problema que los hombres consideraban tener, pero que no podían ver, es decir, al eliminar el cáncer, también se eliminan las preocupaciones. La literatura señala que los hombres en vigilancia activa manifestaron ansiedad en relación con la progresión del cáncer, ya que esta modalidad terapéutica no es definitiva^(15)^. De esta manera, se entiende que el tratamiento definitivo, como la extirpación quirúrgica, puede simbolizar un control más adecuado del cáncer para algunos hombres.

En el DSC E, la cirugía se consideró un procedimiento que extirpaba una parte del cuerpo que tenía problemas, que no funcionaba bien y que podía causar la muerte, lo que demuestra una familiarización con el cáncer y sus consecuencias a través del anclaje. El resultado del estudio corrobora este discurso, ya que los hombres, temiendo la progresión de la enfermedad, vieron en la opción quirúrgica la única manera de eliminar ese miedo, curar el cáncer y prolongar la vida^(16)^.

Además, la cirugía se consideraba una bendición divina, ya que ponía fin al sufrimiento, traía la curación y evitaba la muerte. En consonancia con estos resultados, aquellos que optaron por la extirpación quirúrgica de la próstata relataron alivio por haber liberado sus cuerpos del cáncer y que Dios los había bendecido^(15)^.

En el DCS F, la prostatectomía se consideraba una intervención necesaria ante un tumor maligno que, de forma lenta, podía agravarse y extenderse. Para representar la decisión de someterse a una prostatectomía, utilizaron términos técnicos, como “tumor maligno”, al tiempo que anclaban significados al describir las consecuencias del cáncer. Este resultado concuerda con los hallazgos del estudio, en el que los participantes reconocieron la necesidad de la intervención quirúrgica, aunque ello supusiera pérdidas relacionadas con las funciones sexuales y urinarias, y que, ante este dilema, la opción por la vida era mayor que las consecuencias negativas derivadas de la cirugía^(16)^.

En el DSC K, los participantes alegaron desconocer la importancia de la prostatectomía y se arrepintieron de no haberse sometido a ella antes. Un estudio que evaluó las experiencias de los hombres y el impacto en su bienestar demostró que aquellos que se sometieron a radioterapia y tuvieron una recurrencia del cáncer de próstata creían que la extirpación total de la próstata habría evitado este problema^(17)^.

Este DSC confirma la necesidad de que los profesionales de la salud ofrezcan explicaciones sobre las opciones terapéuticas, sus riesgos y beneficios de manera personalizada^(18)^, respetando los principios de la alfabetización en salud, con un lenguaje que facilite la comprensión, promoviendo la autonomía en el proceso de toma de decisiones sobre la terapia, ya que la aparición de efectos secundarios y la recurrencia del cáncer pueden llevarles a dudar de la eficacia de la opción elegida y de la confianza en los profesionales de la salud^(18,19)^.

En el DSC C, los hombres informaron que la prostatectomía no causó secuelas ni cambios en sus vidas. La eficacia del tratamiento, la ausencia de dolor y la preservación de la función sexual y urinaria pueden estar relacionadas con estas representaciones, ya que, como dicen, estas son consecuencias que pueden afectar a otras personas de su entorno.

Se encontraron percepciones similares en un estudio en el que el participante mencionó que se sentía muy afortunado, ya que, a pesar de presentar disfunción eréctil, gracias a los medicamentos podía mantener relaciones sexuales, a diferencia de otros hombres que conocía^(15)^. En otra investigación, la autopercepción de “alguien que ha tenido cáncer”, asociada a un escaso compromiso con el bienestar general, fue asumida por hombres más jóvenes y sexualmente más activos^(5)^. Así, se entiende que mantener la función sexual, aunque sea con ayuda terapéutica, hace que los hombres representen la cirugía como algo que ha tenido poco o ningún impacto en sus vidas.

En el DSC I, para los participantes, no sirve de nada lamentarse por la prostatectomía, ya que es necesario cuidar la próstata cuando se es más joven, evitando así el cáncer como consecuencia. El resultado de la investigación reveló que los participantes mencionaron que los hábitos nocivos, como el consumo de alcohol y tabaco, causaron el cáncer, y que en la juventud no se piensa en las consecuencias de estos hábitos^(20)^.

En otro estudio, los hombres que habían pasado por una prostatectomía hacía 10 años informaron que el mejor consejo que podían dar a otros hombres era sobre la importancia del cuidado de la salud. Según los autores, esta experiencia fue una forma de que los hombres se conectaran con la masculinidad colectiva^(15,21)^. Ante esto, las experiencias con el cáncer de próstata y la cirugía hicieron que los hombres valoraran los cuidados relacionados con las medidas para la prevención de la enfermedad.

Para los participantes en el presente estudio, someterse a una prostatectomía puede ser motivo de burlas por las secuelas derivadas de la cirugía; sin embargo, no se sintieron avergonzados ante esta realidad. El estigma del cáncer de próstata que impregna el universo simbólico de algunos hombres puede conducir a la vulnerabilidad, haciendo que los hombres que se han sometido a una prostatectomía oculten sus luchas y mantengan en secreto los efectos adversos de la cirugía, lo que culmina en el aislamiento social^(22)^.

Dicho esto, se entiende que los hombres deben superar la vergüenza que impregna las creencias sobre la masculinidad, para así poder obtener el apoyo que necesitan para enfrentar el cáncer y las secuelas derivadas de los procedimientos terapéuticos.

El peso de la representación de la pérdida de la función sexual, evidenciado en el DSC B, llevó a los hombres a cuestionar la relevancia de las explicaciones de los profesionales de la salud, lo que podría orientarlos a no realizar la cirugía. Se encontraron resultados similares en un artículo, en el que los hombres se arrepintieron de haberse sometido a la prostatectomía, tras el conocimiento obtenido a través de la lectura de textos científicos, ya que entendieron que posponiendo el procedimiento podrían tener más tiempo de vida sexual^(15)^. En este discurso se reconoce el peso que representa la pérdida de la función sexual, ya que el conocimiento de este problema podría orientarlos a no realizarse la cirugía.

Para los participantes, la prostatectomía se percibió como una “mutilación”, representación que se basa en la extirpación de una parte del cuerpo y de su función. Los resultados de la investigación indicaron que la alteración de la función eréctil hizo que los hombres se sintieran incapaces de cumplir con su deber como maridos en la relación sexual de la pareja^(16)^. Dicho esto, las esposas deben ser incluidas en la atención, para que puedan discutir, junto con su cónyuge y los profesionales de la salud, los efectos adversos de la prostatectomía^(23)^.

Ante esta realidad, se ha defendido la importancia de la prerhabilitación, que consiste en un enfoque multidisciplinario antes de la intervención quirúrgica, que en la prostatectomía consiste en abordar los posibles efectos secundarios antes de que se manifiesten después de la cirugía. Este enfoque tiene como objetivo mejorar la capacidad funcional del hombre, la resiliencia psicológica y la salud general, aliviando el impacto negativo de la cirugía en la calidad de vida. Entre las intervenciones utilizadas en esta fase se encuentran: actividad física, apoyo de pares (por ejemplo, grupo de hombres con cáncer), entrenamiento de los músculos del suelo pélvico, orientación nutricional y administración de inhibidores de la fosfodiesterasa-5^(24)^. Este enfoque es fundamental para que los hombres puedan reelaborar mejor los efectos adversos derivados de la prostatectomía, motivándolos a buscar recursos para mitigar dichos efectos.

En cuanto al DSC D, los participantes dijeron que después de la cirugía notaron la pérdida del control urinario, lo que exigió cambios en sus hábitos, como la posición para usar el inodoro y el uso de pañales. En este discurso, los participantes se apropian de la imagen de una ducha para anclar representaciones relacionadas con la forma en que entienden el patrón urinario actual.

Se encontraron resultados similares en estudios en los que los hombres informaron que la incontinencia afecta el sueño, ya que los pañales y los absorbentes no son totalmente eficaces para controlar el volumen urinario durante la noche, lo que les impide dormir^(15)^, además de hacer que los hombres eviten las ocasiones sociales^(25)^, se sienten avergonzados al usar baños públicos^(26)^, tienen problemas para viajar, se preocupan por tener mal olor y restringen el consumo de líquidos^(25,27)^. Además, el uso de pañales se señaló como algo que los despersonalizaba, haciendo que los hombres se sintieran como niños^(14)^.

En vista de este contexto, se comprenden los impactos que puede representar la pérdida del control esfinteriano, alterando la forma en que los hombres viven, se comportan y establecen sus relaciones sociales.

En este ámbito, como estrategia para el seguimiento de los hombres con incontinencia, investigadores brasileños desarrollaron la aplicación IUProst®, considerada una tecnología que puede favorecer la atención prestada por los enfermeros, ya que esta aplicación ofrece información sobre cambios en los hábitos de vida y la realización de ejercicios para fortalecer los músculos pélvicos, permitiendo la realización de los ejercicios de forma remota, a través de un teléfono inteligente^(28)^.

En el DSC H, el implante de próstata se representó como una alternativa para sustituir la próstata extirpada. Se deduce que la próstata se representa como un órgano asociado a la capacidad eréctil, de modo que un implante les permitiría recuperar esta función.

Para el sujeto colectivo, el procedimiento quirúrgico se representó como algo para salvar la vida; sin embargo, el impacto negativo de las secuelas modificó la percepción, demostrando que el sentido de la vida no solo está ligado a no morir, sino a cómo se vive.

En este contexto, la enfermería debe buscar enfoques innovadores de atención que contribuyan a mejorar la calidad de vida. Las evidencias de la atención colaborativa demuestran que las habilidades profesionales de enfermería, combinadas con la investigación científica y centradas en las necesidades individuales, fueron eficaces para el desarrollo de planes de atención personalizados para hombres después de la prostatectomía, ya que se evidenció un mayor bienestar emocional, una mejor capacidad de autocuidado y calidad de vida y una reducción de las complicaciones posoperatorias^(29)^.

En el DSC G, la cirugía se percibió como algo desagradable y frustrante, que le hizo pasar por momentos difíciles, causándole malestar físico y psicológico, derivado del procedimiento y también de la palabra cáncer. El estudio demuestra que la representación social del cáncer para las personas adultas en tratamiento oncológico, en un primer momento, impregna significados relacionados con la enfermedad, la tristeza y la muerte, en los que la tristeza se presenta como un sentimiento negativo ante la posibilidad de enfermar y la muerte como una relación directa vinculada al diagnóstico de cáncer^(7)^. En estas circunstancias, es necesario el diálogo y el acompañamiento psicológico, para que los hombres puedan afrontar el tratamiento con más tranquilidad.

Los resultados del estudio corroboran estos hallazgos, ya que los hombres en tratamiento por cáncer de próstata se enfrentan a problemas psicológicos continuos relacionados con el cáncer y su tratamiento, lo que ha tenido un impacto en su autoestima y sus relaciones. También señalaron que la búsqueda de apoyo para sus problemas psicosociales es algo difícil, ya que existe cierta vergüenza a la hora de discutir estas cuestiones^(30)^.

En el DSC J, las representaciones que impregnan el universo consensuado masculino se refieren a la preocupación por la pérdida de la función sexual, como se observa en la literatura, en la que las preocupaciones en torno a los posibles resultados no deseados hicieron que los hombres presentaran incertidumbres sobre qué tratamiento debían elegir, ya que, al mismo tiempo que percibían mejores resultados mediante la cirugía, presentaban incertidumbres en relación con los riesgos de disfunción urinaria y sexual, siendo este el factor central en el proceso de toma de decisiones^(17)^.

Así, se entiende que las representaciones sobre los efectos indeseados de la prostatectomía impregnan el universo consensuado masculino, causando preocupaciones, lo que puede orientar el proceso de toma de decisiones terapéuticas.

Además, informaron que fue un shock y un susto. Se deduce que los hombres representaron la cirugía como algo que los sacude, que los saca de su lugar y que difícilmente vuelven a la “normalidad”. Este lugar puede simbolizar su identidad como hombres sanos, con control de sus cuerpos y funciones. Se reitera la importancia de que los hombres estén informados sobre el procedimiento quirúrgico, ya que la mayoría de los participantes sintieron que no tenían la información necesaria para manejar los efectos secundarios a largo plazo y que no sabían cómo volverían a la “normalidad”, por lo que desconocían los recursos disponibles para manejar sus problemas continuos^(30)^.

Como limitaciones de este estudio, se considera que no fue posible identificar situaciones de remisión de la enfermedad, el tipo de abordaje quirúrgico laparoscópico o abierto y la preservación de los haces neurovasculares, así como datos relacionados con la incontinencia urinaria y la disfunción sexual, lo que podría contribuir a una mejor caracterización de los participantes. Además, las entrevistas no se devolvieron a los participantes, debido al flujo de atención del centro oncológico.

Como contribuciones a la práctica, los resultados revelaron la importancia de la alfabetización en salud para la comprensión de las orientaciones, lo que contribuye a la coparticipación del hombre en las decisiones terapéuticas, así como a la participación de las parejas/esposas en las orientaciones sobre la prostatectomía y sus efectos adversos. Para que estas orientaciones sean eficaces, se deben crear espacios de diálogo que permitan a los hombres superar las barreras de la vergüenza y obtener el apoyo que necesitan.

Las estrategias personalizadas e innovadoras, basadas en el autocuidado apoyado y en la evidencia científica actual, pueden contribuir a aliviar el sufrimiento, mejorar las funciones físicas relacionadas con la sexualidad y la función urinaria, además de la comprensión de la enfermedad y los tratamientos.

## CONSIDERACIONES FINALES

El análisis de las representaciones sociales reveló que, para algunos hombres, la prostatectomía radical se consideraba un procedimiento beneficioso y curativo, una atención médica necesaria, lo que demostraba los beneficios y la satisfacción con el tratamiento. Por otro lado, otros discursos expresaban la prostatectomía como una experiencia relacionada con el sufrimiento, los daños en la función sexual, la virilidad, el control urinario y la relación conyugal. La ausencia de la próstata fue lamentada, ya que sienten que la cirugía les salvó la vida, pero que no están viviendo.

Se sugiere realizar estudios en otros contextos socioculturales y delimitar el tiempo posoperatorio, así como las fases de tratamiento y seguimiento.

## Data Availability

Todo el conjunto de datos que respalda los resultados de este estudio fue puesto a disposición en SciELO Data y puede ser accedido en https://doi.org/10.48331/SCIELODATA.AJLTDE.
